# Enterovirus 71 Infection Shapes Host T Cell Receptor Repertoire and Presumably Expands VP1-Specific TCRβ CDR3 Cluster

**DOI:** 10.3390/pathogens9020121

**Published:** 2020-02-14

**Authors:** Yu-Wen Liao, Bing-Ching Ho, Min-Hsuan Chen, Sung-Liang Yu

**Affiliations:** 1 Department of Clinical Laboratory Sciences and Medical Biotechnology, College of Medicine, National Taiwan University, Taipei 100, Taiwan; d03424001@ntu.edu.tw; 2 Centers of Genomic and Precision Medicine, National Taiwan University, Taipei 100, Taiwan; f94424002@ntu.edu.tw (B.-C.H.); amp542chen@gmail.com (M.-H.C.); 3 Department of Laboratory Medicine, National Taiwan University Hospital, Taipei 100, Taiwan; 4 Institute of Medical Device and Imaging, College of Medicine, National Taiwan University, Taipei 100, Taiwan; 5 Graduate Institute of Pathology, College of Medicine, National Taiwan University, Taipei 100, Taiwan; 6 Graduate Institute of Clinical Medicine, National Taiwan University College of Medicine, Taipei 100, Taiwan

**Keywords:** Enterovirus 71, TCRβ repertoires, VP1, CDR3, pathogenesis

## Abstract

Enterovirus 71 (EV71) has become an important public health problem in the Asia-Pacific region in the past decades. EV71 infection might cause neurological and psychiatric complications and even death. Although an EV71 vaccine has been currently approved, there is no effective therapy for treating EV71-infected patients. Virus infections have been reported to shape host T cell receptor (TCR) repertoire. Therefore, understanding of host TCR repertoire in EV71 infection could better the knowledge in viral pathogenesis and further benefit the anti-viral therapy development. In this study, we used a mouse-adapted EV71 (mEV71) model to observe changes of host TCR repertoire in an EV71-infected central nervous system. Neonate mice were infected with mEV71 and mouse brainstem TCRβ repertoires were explored. Here, we reported that mEV71 infection impacted host brainstem TCRβ repertoire, where mEV71 infection skewed TCRβ diversity, changed VJ combination usages, and further expanded specific TCRβ CDR3 clones. Using bioinformatics analysis and ligand-binding prediction, we speculated the expanded TCRβ CDR3 clone harboring CASSLGANSDYTF sequence was capable of binding cleaved EV71 VP1 peptides in concert with major histocompatibility complex (MHC) molecules. We observed that mEV71 infection shaped host TCRβ repertoire and presumably expanded VP1-specific TCRβ CDR3 in mEV71-infected mouse brainstem that integrated EV71 pathogenesis in central nervous system.

## 1. Introduction

Viral infections are always one of the main challenges in public health. Recently, African swine fever, avian influenza and enterovirus 71 (EV71) impacted pork industries and human health [1,2,3]. In particular, EV71 belonging to the *Picornaviridae* family has become a life-threatening pathogen in the Asia-Pacific region since 1997 [4]. EV71 was first identified in California in 1969, and several outbreaks with dozens of fatalities were reported in Malaysia, Japan, Taiwan, Singapore and China [5]. EV71 infects humans through the fecal-oral route, resulting in typical hand-foot-and-mouth disease, encephalomyelitis, brainstem encephalitis, pulmonary edema, poliomyelitis-like paralysis or even neurological and psychiatric complications [4,6,7]. EV71 infects the central nervous system (CNS) within two to five days after skin or mucosal lesions or fever, and most survivors from CNS damage have long-term neurological sequelae or impaired cognitive function [4]. We found that miR-141, miR-146a and miR-370 play critical roles in EV71 pathogenesis involved in host protein synthesis shutdown, apoptosis and immune escape [8,9,10]. However, other than supportive treatment, there is no effective therapy for treating EV71-infected patients, although an EV71 vaccine has been proved and administered in China routinely that provides promising protection against EV71 infection in infants and young children [11,12]. It is worth noting that a single EV71 genotype was used as the vaccine strain and the cross-neutralization efficacy of each EV71 vaccine should be explored [13].

The adaptive immunity is critical for establishing defense mechanisms against pathogen infections where T cell receptors (TCRs) and B cell receptors (BCRs) are the key molecules to recognize specific antigens [14,15]. The foundation of a mature adaptive immunity is based on the enormous diversities of TCR and BCR repertoires. It has been demonstrated that cellular immunity was more related to the clinical outcomes of EV71-infected subjects than humoral immunity [16]. Besides, several studies indicated that interleukin 1β (IL1β), IL6, IL10, IL13, tumor necrosis factor α (TNFα) or interferon γ (IFNγ) were highly elevated in EV71-infected patients with diverse manifestations, including brainstem encephalitis, poliomyelitis-like syndrome, meningitis and pulmonary edema [17,18]. This evidence suggests that immune responses might contribute to EV71 pathogenesis. However, the role of host TCR repertoire on EV71 pathogenesis, especially in the central nervous system, was not clearly explored.

Next-generation sequencing (NGS) technologies are the powerful tools for genomic studies. NGS technology can provide a huge data set that supports researchers to address complicated biological topics such as epigenetic modification, immune repertoire and metagenomics [19,20,21]. TCRs and BCRs are comprised of variable (V), diversity (D), joining (J), and constant (C) gene segments. TCR and BCR repertoires are highly complicated and diverse due to their V(D)J rearrangement and NGS is required to explore immune repertoire. Most of TCRs consists of α-chain and β-chain which have three complementary determining regions (CDRs), respectively. Among CDRs, CDR1α, CDR1β, CDR2α, and CDR2β are determined to interact with MHC molecules while CDR3α and CDR3β are defined to interact with antigen peptides [22,23]. CDR3β shows greater diversity than CDR3α that suggests CDR3β is the key determinant for antigen recognition [24].

In this study, we utilized mouse-adapted EV71 (mEV71) as the experimental model to observe the changes of host T cell receptor β (TCRβ) repertoire in EV71 infection [9]. TCRβ repertoire in the brainstem of mEV71-infected mice was characterized by NGS technology and the viral protein-specific TCRβ CDR3 clones expansion in mEV71-infected subjects were also identified. Consequently, this could better our knowledge in EV71 pathogenesis especially in the central nervous system and might potentially benefit the anti-viral therapy development.

## 2. Results

### 2.1. EV71 Infection Skewed the Host TCRβ Repertoire

**Figure 1:**
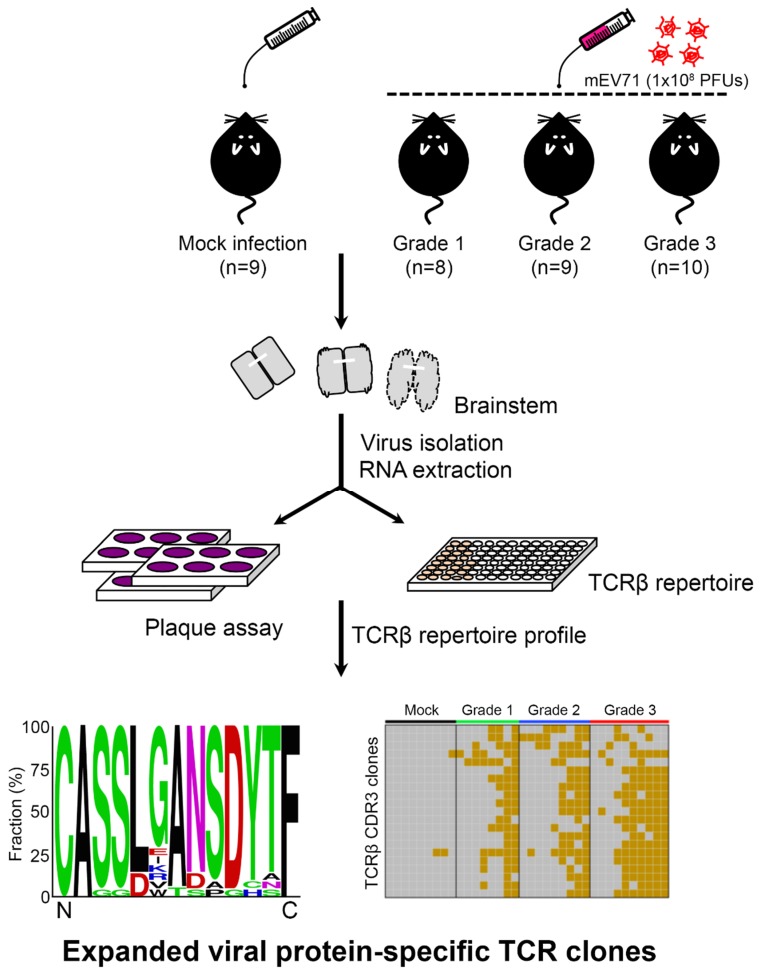
The schematic diagram for exploration of the alteration of host TCRβ repertoire in mEV71 infection. Mice were first infected with 1 × 10^8^ plaque-forming units (PFUs) of mEV71 and grouped by severity assessment (Grade 1, Grade 2, and Grade 3). RNAs obtained from the mouse brainstem were subjected to TCRβ repertoire profiling using iRepertoire technology and next-generation sequencing (NGS). Viruses isolated from the brainstem were titrated by plaque assays. Pair-end sequencing reads were merged and translated to yield TCRβ CDR3 repertoire data. The TCRβ diversities, TCRβ VJ combination usages, and expanded TCRβ CDR3 sequence consensus as well as their prevalence were determined in this study. mEV71: mouse-adapted EV71.

**Figure 2:**
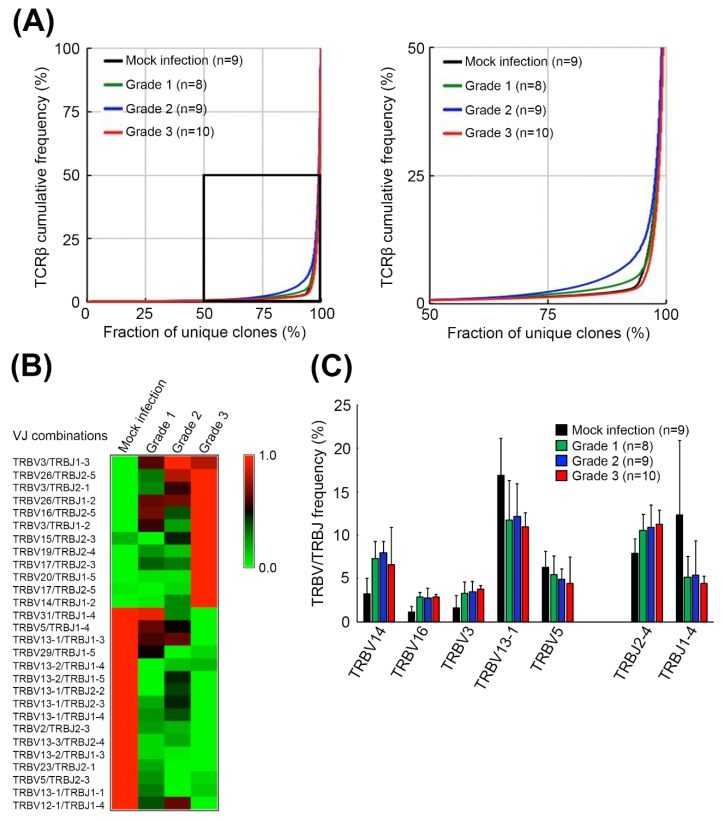
Diversities and VJ combination usages of TCRβ repertoires in the brainstems of mEV71-infected mice. (**A**) The TCRβ diversities in the brainstem of mEV71-infected mice. The TCRβ cumulative frequencies of mice with different symptom severities were plotted. The diversities of the TCRβ repertoire in the brainstem were increased in Grade 1 and Grade 2 but decreased in Grade 3. The right figure is a close view of the rectangular region in the left figure. (**B**) The relative frequencies of VJ combination usages in mice with different symptom severities. The usage of VJ combinations with a significant *p* value of trend test < 0.05 in mEV71 infection was identified. The relative frequencies of 12 VJ combination usages were increased while 16 VJ combination usages were decreased in mEV71 infection. The frequencies presented in the heatmap were scaled by row. (**C**) The TRBV and TRBJ frequencies in mice with different symptom severities. The frequencies of the TRBV and TRBJ gene families with significant alterations (*p* value of trend test < 0.05) in mEV71 infection were identified and illustrated. Five TRBV gene families and 2 TRBJ gene families were differentially expressed with severity grading in mEV71-infected mice compared to those in mock infection group. *p* value of trend test < 0.05.

To investigate the host TCRβ repertoire in EV71 infection, an established mouse-adapted EV71 (mEV71) was used in this study according to our previous report [9]. We infected 5-day-old neonate C57BL/6 mice with 1 × 10^8^ plaque-forming units (PFUs) of mEV71 to generate mEV71-infected mice with different disease severities. The mEV71-infected mice were divided into three groups, from grade 1 to grade 3 (designated G1, G2 and G3 hereafter), based on the severity assessment (Figure 1). The titers of viruses isolated from the brainstem of virus-infected mice in the G2 and G3 groups were significantly higher than those in the G1 group (Table 1). Likewise, the average body weight in the G3 group was significantly decreased compared with that in the mock infection group. Furthermore, the virus titer and body weight loss were significantly positively correlated with the severity of mEV71 infection (*p* value of trend test < 0.001). It is known that virus infection might impact host local or systemic TCRβ repertoires [25]. Hence, the brainstem TCRβ profiles of mock- and virus-infected mice were assessed by iRepertoire (iRepertoire, Inc., Huntsville, AL, USA) and NGS (Figure 1). Paired-end sequencing reads were first merged by the BLAT (BLAST-like alignment tool) algorithm [26], and the merged reads were mapped to the IMGT (ImMunoGeneTics) database [27]. The average of mapped sequencing reads for every severity grade was approximately 1.11 M (Supplementary Table S1). We then evaluated TCRβ sequence diversity in each group, and the cumulative frequencies of TCRβ clone types in the brainstem were calculated [28]. The results showed that TCRβ repertoire diversities in the G1 and G2 groups increased with severity but decreased in the G3 group, indicating that certain TCRβ clone types emerged drastically in the G3 group and gave rise to the worst severity (Figure 2A). Next, to determine whether mEV71 infection influences host TCRβ VJ combination usage, the VJ combination usages were analyzed. Twenty-eight significantly altered VJ combinations were identified and correlated with grading in mEV71 infection (*p* value of trend test < 0.05) (Figure 2B and Supplementary Figure S1). Among 28 altered VJ combinations, 12 VJ combinations were increased and 16 VJ combinations were decreased. We further analyzed the usages of TRBV and TRBJ and identified the TRBV and TRBJ families with significant alteration in mEV71 infection interpreting by trend *p* value (<0.05). The frequencies of three TRBV gene families were significantly increased (TRBV14, TRBV16 and TRBV3) and those of two families, TRBV13-1 and TRBV5, were decreased (Figure 2C). Regarding TRBJ usage, two altered TRBJ gene families were identified; TRBJ2-4 increased and TRBJ1-4 decreased (Figure 2C). Taken together, these data indicated that mEV71 infection influences host TCRβ VJ combination usage, alters clone type diversities and further impacts the TCRβ repertoire. Importantly, alteration of the host TCRβ repertoire was associated with disease severity.

### 2.2. The Expanded TCRβ CDR3 Clones Prevailed in mEV71-Infected Mouse Brainstems

**Figure 3:**
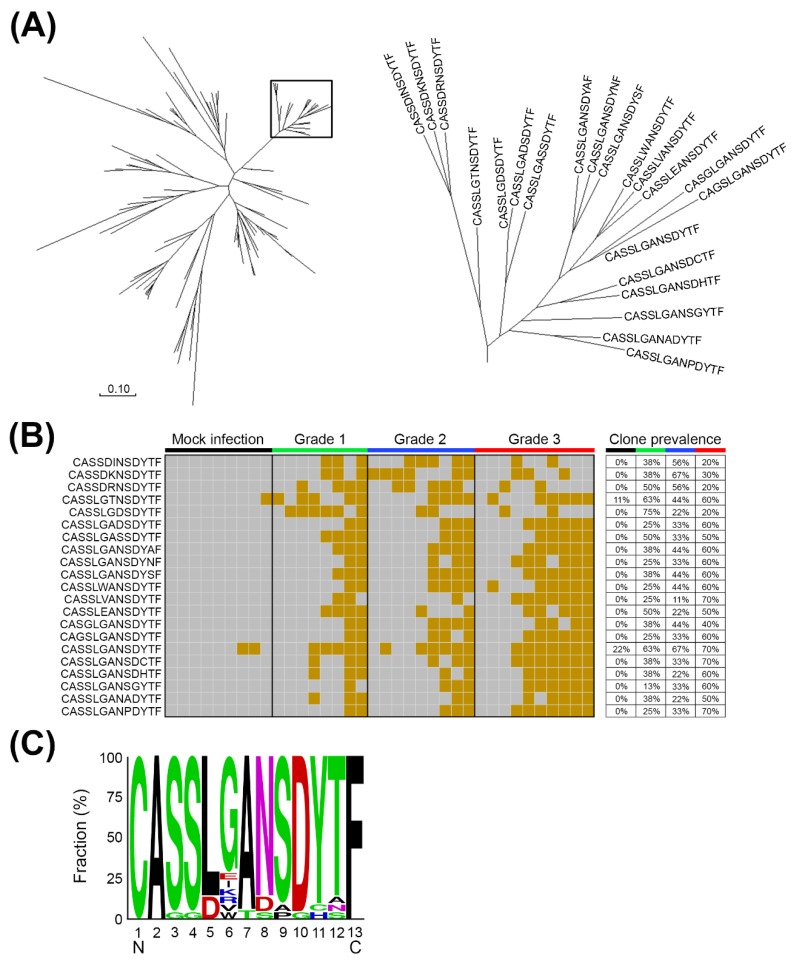
The expanded TCRβ CDR3 clones in the brainstems of mEV71-infected mice. (**A**) The clustering of expanded TCRβ CDR3 clones. The merged sequencing reads were first translated into amino acid sequences, and the amino acid sequences located in the hypervariable CDR3 of the TCRβ chain were used for expanded CDR3 clone identification. A total of 99 TCRβ CDR3 clones which significantly expanded in virus infection groups comparing to the mock infection group were identified, and 21 TCRβ CDR3 clones were clustered, which were circled in the rectangle, by the neighbor-joining method. (**B**) The expanded TCRβ CDR3 clones prevailed with grading among mEV71 infection groups. Twenty-one TCRβ CDR3 clones in the cluster were stimulated in response to mEV71 infection. The brown grid represents a detectable expression level; the gray grid represents an undetectable expression level. (**C**) The core CDR3 sequence of the expanded TCRβ cluster. The CDR3 sequence CASSLGANSDYTF represented the core TCRβ CDR3 clone.

Brainstem involvement is one of the main causes of EV71-induced fatalities; therefore, we speculated that TCRβ CDR3 alterations might be involved in viral pathogenesis. To identify the potential expanded TCRβ CDR3 clones in response to EV71 infection, the amino acid sequences of the hypervariable CDR3 of the TCRβ chain were analyzed. TCRβ sequencing reads were first translated into six frames to acquire corresponding amino acid epitopes, and the TCRβ CDR3 clones with significant alteration in mEV71 infection comparing to those in mock infection were identified. A total of 99 TCRβ CDR3 clones with significant alteration in the brainstem were identified, and potential expanded TCRβ CDR3 clusters were generated by the neighbor-joining method. As shown in Figure 3A, the cluster circled in the rectangle containing 21 TCRβ CDR3 clones was selected for further analysis due to the slower rate of amino acid substitution within the cluster. Hence, we hypothesized that this specific TCRβ CDR3 clone cluster might be stimulated by the viral peptides. To address our hypothesis, we determined the prevalence of the 21 TCRβ CDR3 clones in the brainstems of mEV71-infected mice in the cluster. The results suggested that the 21 TCRβ CDR3 clones were stimulated in response to mEV71 infection, particularly those in the G3 group (Figure 3B). The consensus TCRβ CDR3 sequence, CASSLGANSDYTF, was deduced from the 21 TCRβ clones by WebLoGo (Figure 3C) [29,30].

### 2.3. The Expanded TCRβ CDR3 Clones Were Elicited by EV71 Viral Protein 1

EV71 viral protein 1 (VP1) is one of the major antigenic targets for anti-EV71 vaccines and is also regarded as an antigenic determinant for T cell responses [16,31]. VP1 is located on the outer side of the capsid, used for EV71 genotyping and considered the key epitope of EV71 [16,31]. To investigate the importance of the CASSLGANSDYTF TCRβ CDR3 clone for the binding of EV71 VP1, we conducted in silico viral peptide binding prediction. First, the full-length amino acid sequence of VP1 protein of EV71 strain Tainan/5746/9 (AF304457.1) was used to predict the potential cleaved EV71 VP1 peptides presented by major histocompatibility complex (MHC) I and MHCII (Supplementary Tables S2 and S3). We then selected the potential viral peptides by considering the number of binding (NB) to mouse MHC of greater or equal to two for further analysis. The NB of four MHCI-favored (FTYMRFDAEFTF, LAWQTATNPSVF, IYMRMKHVRAWI, and SFFSRAGLVGEI) and one MHCII-favored cleaved VP1 peptides (VSRALTRALPAPTGQ) of mEV71 were greater than or equal to two (Supplementary Tables S2 and S3). In addition to EV71 VP1, it was reported that EV71 VP2 showed a broad distribution of immunogenic peptides that could dominate T cell responses against EV71 [32]. Therefore, we analyzed the potential cleaved EV71 VP2 epitopes presented by mouse MHCI and MHCII (Supplementary Tables S4 and S5). Based on the same selection criteria, four cleaved EV71 VP2 peptides (CGYSDRVAQLTI, CNASKFHQGALL, DSALNHCNFGLL, and SALNHCNFGLLV) favored mouse MHCI with a NB equal to two, while none of the cleaved VP2 peptides favored mouse MHCII with a NB greater than or equal to two (Supplementary Tables S4 and S5).

**Figure 4:**
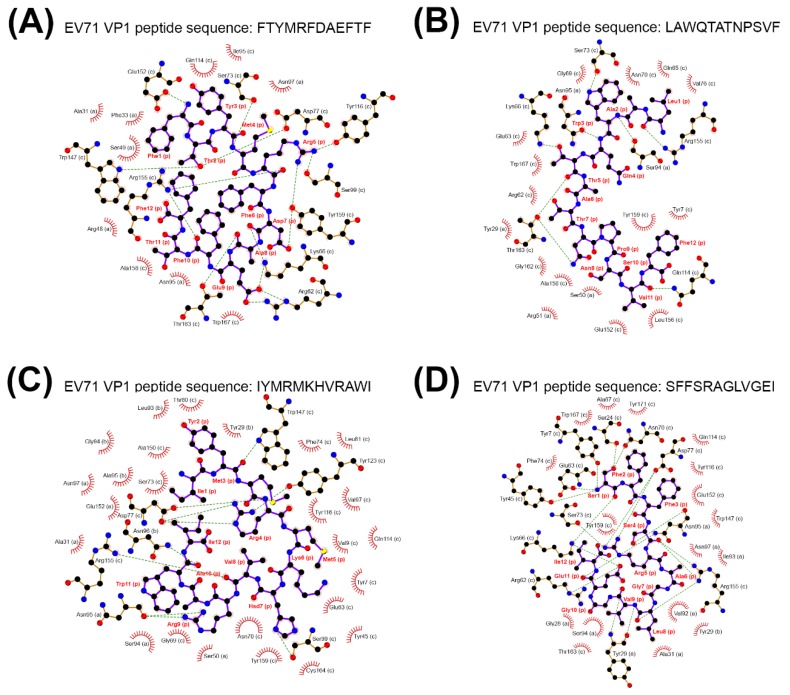
The binding complex between TCRβ CDR3, EV71 VP1 peptide and major histocompatibility complex (MHC) class I. The binding complexes composed of TCRβ CDR3 CASSLGANSDYTF, MHC class I molecule and cleaved EV71 VP1 peptide were illustrated, where distances between interacting side chains were not considered. The binding complexes were composed of the cleaved EV71 VP1 peptides FTYMRFDAEFTF (**A**), LAWQTATNPSVF (**B**), IYMRMKHVRAWI (**C**), and SFFSRAGLVGEI (**D**). The green dashed line represents hydrogen binding; the red spike represents hydrophobic interactions; the alphabet in parenthesis represents the chain ID in the protein complex; the ball and stick in black and purple represent the cleaved EV71 VP1 peptide.

Regarding the NB of each cleaved viral peptide with a NB greater than or equal to two, the identified cleaved VP1/VP2 peptides fitted to the criterion were adapted to binding potential prediction with the TCRβ-MHCI/II complex containing a CASSLGANSDYTF CDR3 sequence. The ligand-binding prediction between the TCRβ-MHC protein complex and cleaved EV71 VP1 peptides was carried out by ClusPro [33,34,35]. The results indicated that the binding potential highly favored MHCI-VP1 peptide complexes but not MHCII-VP1 peptide complexes [36,37] (Table 2). For EV71 VP2 peptides, only three MHCI-favored viral peptides performed binding potential with the TCRβ-MHCI complex (Supplementary Table S6). Collectively, four MHCI-favored cleaved VP1 peptides and three out of four MHCI-favored cleaved VP2 peptides showed binding potential with the TCRβ-MHCI complex (Table 2 and Supplementary Table S6), while none of MHCII-favored cleaved VP1 or VP2 peptides performed binding potential with TCRβ-MHCII complex. We then determined the ligand-binding energies between cleaved EV71 VP1 peptides and the TCRβ-MHCI complex. The four MHCI-favored cleaved VP1 peptides of mEV71 (FTYMRFDAEFTF, LAWQTATNPSVF, IYMRMKHVRAWI, and SFFSRAGLVGEI) were aligned to EV71 strain Tainan/5746/9. The alignment showed that the amino acid sequences of all cleaved mEV71 VP1 peptides were identical to EV71 strain Tainan/5746/9 (Supplementary Figure S2). The binding energies between the EV71 VP1 peptide and the TCRβ-MHCI complex were predicted by DOCK6 since the scores implemented from the ClusPro service cannot be used to compare the binding energy among peptide-bound TCRβ-MHCI complexes (https://cluspro.bu.edu/help.php). The results suggested that stable binding was predicted between four cleaved EV71 VP1 peptides and the CASSLGANSDYTF-bearing TCRβ-MHCI complexes (Table 3) [36,37]. To scrutinize the spatial arrangements of the CDR3 loop with the viral peptide bound, force-field molecular dynamics were applied to the four stable peptide-bound protein complexes. Our analyses suggested that the CDR3 loop secures if not interacts with the viral peptides in the binding groove. In other words, the CDR3 loop functions as a gate to prevent the viral peptide from escaping and, in a few complexes, exerts hydrophobic interactions on the amino acids of the viral peptide (Figure 4 and Supplementary Figure S3).

### 2.4. Scrambled CDR3 Sequences Disturbed the Stable Binding of TCRβ-MHC-Viral Peptide Complex

A scrambled CASSLGANSDYTF CDR3 sequence was used to substitute the CDR3 loop of the TCRβ protein and to measure whether the binding stability between the scrambled TCRβ-MHCI complexes and viral peptides was disturbed. A DOCK6 prediction showed that the scrambled CDR3 sequences reduced the energy of viral peptide binding [38], caused a confirmation change and finally destabilized the binding between CDR3 and EV71 VP1 peptides (Table 4 and Supplementary Figure S4).

LIGPLOT [39] visually suggested that scrambled CDR3 sequences were associated with drastic conformational changes in the binding groove, giving rise to binding instability (Supplementary Figure S4). Nonetheless, computationally, we are unable to distinguish the causality between conformational changes and binding instability. For example, when the viral peptide FTYMRFDAEFTF bound to the CASSLGANSDYTF-containing TCRβ-MHC protein complex, LIGPLOT indicated an environment with side chains in proximity between the viral peptide and the protein complex (Figure 4 and Supplementary Figure S3). In contrast, once the scrambling took place a huge cavity appeared, resulting in weakened hydrogen-bonding energy and abolishment of several hydrophobic interactions. These phenomena undermined the stability and, accordingly, were reflected in Grid scores using DOCK6 (Table 4 and Supplementary Figure S4). Taken together, these data showed that the stable binding between the identified expanded CDR3 clone and viral peptide-MHCI might be highly associated with a specific combination of amino acids in the CDR3 loop.

## 3. Discussion

T cell-mediated antiviral immunity is principally composed of T cells, MHC molecules and viral peptides, wherein T cells play a pivotal role in antigen recognition and immune attack [40]. Virus infections have been demonstrated to shape the host T cell receptor repertoire, including those by influenza virus, human cytomegalovirus (HCMV) and Epstein-Barr virus (EBV) [25,41,42,43]. To the best of our knowledge, this is the first report exploring the impacts of the host TCRβ repertoire in EV71 infection. We determined TCRβ repertoire diversities in the brainstem of mEV71-infected mice, and the diversities were approximately increased with severity grading but were dominated by expanded TCRβ CDR3 clones in the severest group (Figure 2A and Figure 3B). Shifrut et al. reported a more skewed TCRβ repertoire in old mice (17–20 months old) than in young mice (6–8 weeks old) [28]. The neonate mice showed the most skewed repertoire compared to that of either young or old mice, although virus infection increased TCRβ repertoire diversities (Figure 2A). Moreover, the diversity of the splenic TCRβ repertoire was greatly reduced compared with that of the bone marrow-derived TCRβ repertoire in the same-age mice [28]. This evidence indicated that TCRβ repertoires are sensitive to age, repertoire location and stimulation (Figure 2A) [28,44].

EV71 infection is an important public health problem with life-threatening impacts in southeastern Asia. To solve this problem, numerous studies have focused on vaccine development in the past decade [45,46,47,48,49], and inactivated EV71 vaccines, providing an invaluable gift for children, were developed successfully [11,12,50]. Several EV71 genotypes such as C4a, B5, C2 were circulating in the Asia-Pacific Region in the past decade and EV71 genotypes might be the key factor to affect vaccine cross-neutralization [51,52,53,54,55]. Hence, it would be further investigated whether EV71 vaccines developed using a single EV71 strain could provide strong cross-protection against other EV71 strains. Although EV71 vaccine evaluation has been completed in phase III clinical trials and approved in China, there is still no effective therapy for treating EV71-infected patients other than supportive treatment [11,12,50]. It is well-documented that highly elevated proinflammatory cytokines, such as IL1β, IL6, IL10, IL13, TNFα or IFNγ, are detected in cases of EV71-infected patients with severe manifestations as well as in fatal cases [17,18]. T cell-mediated immunity and proinflammatory cytokines are considered attackers against virus infections, while an uncontrollable immune reaction is stimulated in EV71 infection and even in other enterovirus infections to cause severe manifestations [5,17,18,56]. Therefore, regulating and suppressing elevated proinflammatory cytokines might be the key to protecting virus-infected subjects from such severe manifestations, at least in part. The skewed TCRβ repertoire was observed in our study and, to our best knowledge, that is one of the potential reasons to explore why elevated proinflammatory cytokines were found in EV71-infected subjects.

Multiple sclerosis (MS) is an immune-mediated demyelinating disease in which the patient’s immune system destroys the myelin sheath and causes disability and neurodegeneration [57]. Natalizumab, an FDA-approved monoclonal antibody drug for MS, serves as an immunosuppressive agent to block activated lymphocytes from crossing the blood-brain barrier and significantly reduces MS-caused disability [58]. We identified an expanded TCRβ CDR3 clone cluster in mEV71-infected mice and characterized the core TCRβ CDR3 clone harboring the CASSLGANSDYTF CDR3 sequence (Figure 3). As a strategy against MS, manipulation of such expanded TCRβ CDR3 clones by neutralization antibodies or specific inhibitors might be considered as a new approach for controlling EV71 infection [59,60].

In summary, we have determined the impacts of EV71 infection on the host TCRβ repertoire wherein EV71 infection altered host TCRβ diversity, changed VJ combination usages and expanded viral protein-specific TCRβ CDR3 clones. Bioinformatics analysis and molecular modeling speculated that EV71 VP1 peptides would be presented by MHC class I to stimulate specific TCRβ CDR3 clone expansion. Our findings suggested EV71 infection could skew host TCRβ repertoire and also expand VP1-specific TCRβ CDR3 clones. Although these results may not adapt to other EV71 infection mouse models directly [61,62,63] and could not completely reflect natural situations in human beings due to the limited experimental mouse model and bioinformatic prediction, these findings could partly broaden our knowledge in EV71 pathogenesis and provide an insight in the anti-viral therapy development potentially via manipulating expanded TCRβ CDR3 clones in virus infection.

## 4. Materials and Methods

### 4.1. Ethics Statement

The National Taiwan University College of Medicine and College of Public Health Institutional Animal Care and Use Committee (IACUC) approved all animal protocols with the identification number 20140264. All animal experiments were in strict accordance with the Guidebook for the Care and Use of Laboratory Animals, 3rd Ed., 2007, published by The Chinese-Taipei Society of Laboratory Animal Sciences.

### 4.2. mEV71 Propagation

mEV71 was established referring to a report published by Wang, Y. F. in 2004 [64]. mEV71 was generated after four serial passages in neonate mice started from parental human EV71. Parental human EV71 was injected intraperitoneally and next generation mEV71, called 1st mEV71, was isolated from neonate mice brainstem tissue at 3 days post-infection (d.p.i.). The isolated 1st mEV71 was then propagated in RD cells, a human rhabdomyosarcoma cell line, with serum-free condition. The passage procedures were repeated for four times as our previous report [9]. Aliquots of viral stocks were stored at −80 °C.

### 4.3. RNA Extraction

Total RNAs of the brainstem from each mouse were extracted by GeneJET^TM^ RNA Purification Kit (Fermentas, Waltham, Massachusetts, USA) according to manufacturer’s protocol. Briefly, tissues obtained from mice were first homogenized by TissueLyser LT (Qiagen, Hilden, Germany) and the lysates were then reacted with lysis buffer containing β-mercaptoethanol and proteinase K. The lysates were transferred into GeneJET RNA purification column for RNA binding and washed with washing buffer. Total RNAs were eluted with nuclease-free water and applied to TCRβ iRepertoire library construction.

### 4.4. Plaque Assay

EV71 plaque assays were carried out in triplicate in 6-well plates. RD cells were infected with 100 μL/per well of diluted viral stocks. After 1 h absorption, the monolayer cells were washed with phosphate buffered saline (PBS) and incubated for 3 days in 0.3% agarose medium overlay. Cells were fixed with formaldehyde and stained with crystal violet. Plaques were counted.

### 4.5. mEV71 Infection

C57BL/6 mice were provided by the Knockout Mouse Core Laboratory of National Taiwan University Center of Genomic Medicine, housed in specific pathogen-free animal rooms, and treated according to guidelines from the National Taiwan University College of Medicine and College of Public Health IACUC. For mEV71 inoculation, five-day-old wild-type C57BL/6 mice housed in the same cage were infected with 1 × 10^8^ plaque forming units (PFUs) of mEV71 through the oral route (Figure 1). Mice in mock infection group were fed with culture medium. The animals were monitored hourly (from 7:00 a.m. to 9:00 p.m.) for clinical signs and mortality and the mice with events such as death occurred out of the interval (from 7:00 a.m. to 9:00 p.m.) were excluded. The mouse was divided into the specific group according to severity assessment described as follows [9]. All mouse tissues were obtained from scarified mice and further assayed for TCRβ repertoire library construction and plaque assay.

### 4.6. Severity Assessment

Mice infected with mEV71 or fed with culture medium were sacrificed at 5 d.p.i. and separated into four groups. The mice were monitored from 7:00 a.m. to 9:00 p.m. daily for clinical signs and mortality and the mouse brainstems were obtained from scarified mice with significant clinical illness signs or death or at 5 d.p.i. if mice without any significant illness signs. The Grade 1 group consisted of mEV71-infected mice which had no signs of serious illness or physical impairment at 5 d.p.i. The Grade 2 group consisted of mEV71-infected mice which were alive but with low vitality or mobility but no paralysis observed at 5 d.p.i. The Grade 3 group consisted of mEV71-infected mice with paralysis in limbs or death before or at 5 d.p.i. The body weights of mice were recorded at sacrifice or at 5 d.p.i. but not as a criterion for severity grade assignment.

### 4.7. TCRβ Library Preparation and NGS

Total RNAs extracted from the brainstems were applied to iRepertoire TCRβ library preparation according to manufacturer’s instructions (iRepertoire, Inc.). Briefly, a two-step PCR reaction was used to amplify the immune repertoire. Template RNAs were combined with iRepertoire primer mix including Fout, Fin, Rout and Rin, RT-PCR enzyme mix, dNTP and buffer and then reacted with the PCR cycling as following: 50 °C for 40 min for reverse transcription reaction, 95 °C for 15 min and 94 °C for 30 s, 60 °C for 5 min and 72 °C for 30 s for 10 cycles; 94 °C for 30 s and 72 °C for 3 min for another 10 cycles, and 72 °C for 10 min. In the first PCR, gene-specific primers targeting to the V or C regions were used. The forward primers, Fout and Fin, are located in the V region while the reverse primers, Rout and Rin, are located in the C region. The Fin and Rin primers were conjugated with sequencing adaptors B and A as universal sequences for second PCR annealing, respectively. The second PCR is carried out using universal primers B and A. After amplification, PCR products were adapted to gel purification and the resulting products were sequenced on MiSeq with MiSeq Reagent Kit v3 (600-cycle) (Illumina, San Diego, CA, USA).

### 4.8. NGS Data Processing

Each pair of 300 bp paired-end reads were merged to produce a single long read if BLAT found an overlap whose sequence identity was > 95%, and whose base quality of each nucleotide was > Q20 [26]. Next, the segments of Vβ and Jβ region were identified using BLAT against the IMGT repertoire for *Mus* musculus (Release 201827-0) [65]. Nucleotide sequences immediately upstream from Vβ genes and downstream from Jβ genes were excised for barcode identification. Properly barcoded sequences were kept for CDR3 analysis.

### 4.9. Identification of mEV71 Infection-Related CDR3 Clones

The TCRβ CDR3 region was recognized following the protocol established by the IMGT collaboration [65]. Sequences that did not meet the standard mentioned in the protocol [65] or that contained stop codons after translation were removed.

The read count of each TCRβ CDR3 clone in a mouse was first normalized against total corresponding read count to obtain the expression level of each CDR3 clone. To identify mEV71 infection-related CDR3 clones, the expression levels were converted to 1, if there was a detectable expression level, or 0, if there was no detectable expression level. Fisher’s exact test was applied to calculate the *p* values of each TCRβ CDR3 clone between mEV71-infected and mock infection groups. Ninety-nine clones were identified with *p* value < 0.05. CDR3 sequences are aligned by the clustal omega algorithm of the European Molecular Biology Laboratory (EMBL, https://www.ebi.ac.uk/Tools/msa/clustalo/) and then the phylogenetic tree is constructed by the neighbor-joining method.

### 4.10. Ligand-Binding Prediction

Full EV71 VP1 and VP2 protein sequences, from the mRNA of Tainan/5746/98 strain under the accession number of AF304457, were applied to the NetMHCpan 2.4 and the NetMHCIIpan 3.0 servers [33,34,35]. Based on the number of binding (NB) to mouse MHC greater or equal to 2, the cleaved viral peptides from each server were identified for further analysis. The crystal structure of TCRαβ-MHCI protein complex was adapted from that of BM3.3 ScFV TCR in complex with the PBM8-H-2KBM8 MHCI molecule (PDB: 2OL3). Likewise, the crystal structure of MHCII protein was from that of mouse MHC class II I-Ab/3K peptide complexed with mouse TCR B3K506 (PDB: 3C5Z). The 3D models of cleaved viral peptides and the TCRβ-MHCI/II protein complex were uploaded to the ClusPro 2.0 server for binding prediction, and considered binding occurring if two or more models suggested viral peptides being bound in the binding groove [36,37]. Nonetheless, due to the drawback where the binding scores in the ClusPro did not truly reflect the energy occurring during the peptide binding, the rigid-body docking was carried out using DOCK6 to assess binding affinity [66]. To elucidate the specificity of CASSLGANSDYTF, the randomly scrambled amino acid sequences by a word scramble generator (https://www.superteacherworksheets.com/generator-word-scramble.html) were used to substitute ASSLGANSDYT in the TCRβ CDR3 molecule of the TCRβ-MHCI/II complexes using Modeller [67].

### 4.11. Molecular Dynamics Analysis

The following protocol was adapted from that by M. H. Chen et al. [68]. Homology modeling of TCRβ protein fused with desired CDR3β sequence was conducted using SWISS-MODEL webserver [69,70,71], and the missing hydrogen atoms were patched using MolProbity4 [72,73]. Each complete homology model was minimized for 4000 steps using Nanoscale Molecular Dynamics (NAMD) [74] and solvated with water molecules (TIP3) using Visual Molecular Dynamics (VMD) [75]. The fully solvated system was 80 Å × 80 Å × 125 Å in size. All molecular dynamic simulations were carried out with full Particle Mesh Ewald (PME) calculations for electrostatic interactions. To ensure that the system was relaxed systematically, simulations were carried out using a Langevin bath as follows:  25 ps with the protein fixed, 25 ps with the entire protein harmonically constrained, 25 ps with the peptide backbone harmonically constrained, and 25 ps with α-carbons harmonically constrained. NPT ensemble, constant pressure and temperature, simulation was then performed for 2 ns. The binding of the ligand to the final homology model was visualized using UCSF Chimera [76].

### 4.12. Statistical Analysis

According to sequencing reads merged by the BLAT algorithm and mapped to the IMGT database, TCRβ VJ combination usages were estimated by read counts normalized with sequencing throughput in 4 different groups. In order to identify the TCRβ VJ combination usages associated with different severity groups, simple liner regression was applied to estimate *p* values of the liner trend of usages among groups. Cut-point of the *p* value was 0.05. Further, the linear trend of the usages of TRBV and TRBJ individually were also evaluated.

Among TCRβ VJ combinations with significantly linear tread in usages across 4 different groups, averages of TCRβ VJ combination usages in groups were scaled and depicted by heatmap.

## Figures and Tables

**Table 1:** Biological significances between each severity classified group.

	Severity Grade				*p* Value of Trend Test
	Mock Infection	Grade 1	Grade 2	Grade 3	
Mice No.	9	8	9	10	
Averaged viral titer (log_10_ PFUs/g)	−	3.28 ± 1.68	5.83 ± 0.34	6.41 ± 0.71	<0.001
Viral titer significance (*p* value) ^a^	−	−	<0.001	<0.001	
Average body weight (g)	4.87 ± 0.55	4.16 ± 1.01	3.89 ± 0.90	3.52 ± 0.60	<0.001
Body weight significance (*p* value) ^b^	−	0.2489	0.0532	0.0032	

^a^ Each viral titer significance was compared against Grade 1 group. ^b^ Each body weight significance was compared against Mock infection group.

**Table 2:** The predicted binding potential between a cleaved EV71 VP1 peptide and TCRβ-MHCI/II complex containing a CASSLGANSDYTF CDR3 sequence.

MHC Presenting EV71 VP1 Peptide	Binding Energy
**MHC Class I-EV71 VP1 Peptide**	
FTYMRFDAEFTF	−1193.3 ^a^
LAWQTATNPSVF	−924
IYMRMKHVRAWI	−872.3
SFFSRAGLVGEI	−881.4
**MHC Class II-EV71 VP1 Peptide**	
VSRALTRALPAPTGQ	−

^a^ The scores, implemented from ClusPro service, are not suitable to compare the binding energy among peptide-bound TCRβ-MHCI/II complexes.

**Table 3:** Predicted binding energies in silicon between a cleaved EV71 VP1 peptide and the TCRβ-MHCI complex.

	Cleaved EV71 VP1 Peptide ^a^			
	FTYMRFDAEFTF	LAWQTATNPSVF	IYMRMKHVRAWI	SFFSRAGLVGEI
HA_RMSDm	0.39	0.17	0.93	0.60
Grid score	−180.63	−164.19	−118.01	−155.11
Grid_vdw	−151.80	−153.65	−96.85	−117.20
Grid_es	−28.83	−10.54	−21.16	−37.91
Int. energy	89.50	63.30	120.48	86.70

^a^ The score indicates the binding energy between the peptide and the TCRβ-MHCI complex.

**Table 4:** The predicted binding energy between a cleaved EV71 VP1 peptide and a scrambled CDR3 sequence.

Cleaved EV71 VP1 Peptide	CASSLGANSDYTF CDR3 Scramble	HA_RMSDm	Grid Score	Grid_vdw	Grid_es	Int. Energy ^b^
FTYMRFDAEFTF	CASSLGANSDYTF	0.39	−180.63	−151.80	−28.83	89.50
	CANSGLSSDAYTF	1.45	483.15	486.10	−2.96	260.33
	CGAYLANSSSDTF	0.51	−117.71	−80.91	−36.81	97.31
	CGSNALYSTDASF	5.88	−14.21	−20.92	6.71	86.97
LAWQTATNPSVF	CASSLGANSDYTF ^a^	0.17	−164.19	−153.65	−10.54	63.30
	CLATGNSAYDSSF	1.37	9095.05	9114.54	−19.49	1037.36
	CLSYATASNGSDF	0.42	−137.52	−130.43	−7.09	84.10
	CTSNGAASSDLYF	2.80	58013.92	58035.42	−21.50	1905.18
IYMRMKHVRAWI	CASSLGANSDYTF	0.93	−118.01	−96.85	−21.16	120.48
	CAALYTDSSGSNF	4.30	1423.73	1409.09	14.63	157.11
	CLTAAGNDYSSSF	0.60	−65.99	−41.48	−24.51	142.61
	CSTSNDSAYLGAF	0.50	166.10	199.76	−33.66	114.02
SFFSRAGLVGEI	CASSLGANSDYTF	0.60	−155.11	−117.20	−37.91	86.70
	CAADGLNSSSTYF	0.46	124.89	162.56	−37.67	85.43
	CASNGSTASLDYF	0.46	−43.34	3.89	−47.22	82.62
	CNSYSALTGDASF	0.54	750.47	778.18	−27.71	81.88

^a^ CASSLGANSDYTF serves as the original form of the identified CDR3 clone. ^b^ Int. Energy represents internal energy that could reduce the occurrence of internal clashes during the torsional optimization.

## Supplementary Materials

The following are available online at https://www.mdpi.com/2076-0817/9/2/121/s1, Figure S1: Differential usages of VJ combinations in brainstems of mEV71-infected mice, referred to Figure 2; Figure S2: Amino acid sequences of cleaved mEV71 VP1 peptides; Figure S3: The binding complex between TCRβ CDR3, EV71 VP1 peptide and MHC class I referred to Figure 4; Figure S4: The binding complex between scrambled TCRβ CDR3, cleaved EV71 VP1 peptide and MHC class I, referred to Figure 4; Table S1: The statistics of mapped sequencing reads in each severity-classified group; Table S2: Potential cleaved EV71 VP1 epitopes presented by MHCI according to mouse alleles; Table S3: Potential cleaved EV71 VP1 epitopes presented by MHCII according to mouse alleles; Table S4: Potential cleaved EV71 VP2 epitopes presented by MHCI according to mouse alleles; Table S5: Potential cleaved EV71 VP2 epitopes presented by MHCII according to mouse alleles; Table S6: The predicted binding potential between a cleaved EV71 VP2 peptide and TCR-MHCI/II complex containing a CASSLGANSDYTF CDR3 sequence.

## References

[1] Yi E.J., Shin Y.J., Kim J.H., Kim T.G., Chang S.Y. (2017). Enterovirus 71 infection and vaccines. Clin. Exp. Vaccine Res..

[2] Sivanandy P., Zi Xien F., Woon Kit L., Tze Wei Y., Hui En K., Chia Lynn L. (2018). A review on current trends in the treatment of human infection with H7N9-avian influenza A. J. Infect. Public Health.

[3] Sanchez-Cordon P.J., Montoya M., Reis A.L., Dixon L.K. (2018). African swine fever: A re-emerging viral disease threatening the global pig industry. Vet. J..

[4] Chang L.Y., Huang L.M., Gau S.S., Wu Y.Y., Hsia S.H., Fan T.Y., Lin K.L., Huang Y.C., Lu C.Y., Lin T.Y. (2007). Neurodevelopment and cognition in children after enterovirus 71 infection. N. Engl. J. Med..

[5] Solomon T., Lewthwaite P., Perera D., Cardosa M.J., McMinn P., Ooi M.H. (2010). Virology, epidemiology, pathogenesis, and control of enterovirus 71. Lancet Infect. Dis..

[6] Huang C.C., Liu C.C., Chang Y.C., Chen C.Y., Wang S.T., Yeh T.F. (1999). Neurologic complications in children with enterovirus 71 infection. N. Engl. J. Med..

[7] Chang L.Y., Huang Y.C., Lin T.Y. (1998). Fulminant neurogenic pulmonary oedema with hand, foot, and mouth disease. Lancet.

[8] Chang Y.L., Ho B.C., Sher S., Yu S.L., Yang P.C. (2015). miR-146a and miR-370 coordinate enterovirus 71-induced cell apoptosis through targeting SOS1 and GADD45beta. Cell. Microbiol..

[9] Ho B.C., Yu I.S., Lu L.F., Rudensky A., Chen H.Y., Tsai C.W., Chang Y.L., Wu C.T., Chang L.Y., Shih S.R. (2014). Inhibition of miR-146a prevents enterovirus-induced death by restoring the production of type I interferon. Nat. Commun..

[10] Ho B.C., Yu S.L., Chen J.J., Chang S.Y., Yan B.S., Hong Q.S., Singh S., Kao C.L., Chen H.Y., Su K.Y. (2011). Enterovirus-induced miR-141 contributes to shutoff of host protein translation by targeting the translation initiation factor eIF4E. Cell Host Microbe.

[11] Zhu F., Xu W., Xia J., Liang Z., Liu Y., Zhang X., Tan X., Wang L., Mao Q., Wu J. (2014). Efficacy, safety, and immunogenicity of an enterovirus 71 vaccine in China. N. Engl. J. Med..

[12] Li R., Liu L., Mo Z., Wang X., Xia J., Liang Z., Zhang Y., Li Y., Mao Q., Wang J. (2014). An inactivated enterovirus 71 vaccine in healthy children. N. Engl. J. Med..

[13] Mao Q.Y., Wang Y., Bian L., Xu M., Liang Z. (2016). EV71 vaccine, a new tool to control outbreaks of hand, foot and mouth disease (HFMD). Expert Rev. Vaccines.

[14] Kwak K., Akkaya M., Pierce S.K. (2019). B cell signaling in context. Nat. Immunol..

[15] Courtney A.H., Lo W.L., Weiss A. (2018). TCR Signaling: Mechanisms of Initiation and Propagation. Trends Biochem. Sci..

[16] Chang L.Y., Hsiung C.A., Lu C.Y., Lin T.Y., Huang F.Y., Lai Y.H., Chiang Y.P., Chiang B.L., Lee C.Y., Huang L.M. (2006). Status of cellular rather than humoral immunity is correlated with clinical outcome of enterovirus 71. Pediatric Res..

[17] Lin T.Y., Hsia S.H., Huang Y.C., Wu C.T., Chang L.Y. (2003). Proinflammatory cytokine reactions in enterovirus 71 infections of the central nervous system. Clin. Infect. Dis. Off. Publ. Infect. Dis. Soc. Am..

[18] Wang S.M., Lei H.Y., Huang K.J., Wu J.M., Wang J.R., Yu C.K., Su I.J., Liu C.C. (2003). Pathogenesis of enterovirus 71 brainstem encephalitis in pediatric patients: Roles of cytokines and cellular immune activation in patients with pulmonary edema. J. Infect. Dis..

[19] Schirmer M., Garner A., Vlamakis H., Xavier R.J. (2019). Microbial genes and pathways in inflammatory bowel disease. Nat. Rev. Microbiol..

[20] Nakagawa H., Fujita M. (2018). Whole genome sequencing analysis for cancer genomics and precision medicine. Cancer Sci..

[21] Zhuang Y., Zhang C., Wu Q., Zhang J., Ye Z., Qian Q. (2019). Application of immune repertoire sequencing in cancer immunotherapy. Int. Immunopharmacol..

[22] Fields B.A., Ober B., Malchiodi E.L., Lebedeva M.I., Braden B.C., Ysern X., Kim J.K., Shao X., Ward E.S., Mariuzza R.A. (1995). Crystal structure of the V alpha domain of a T cell antigen receptor. Science.

[23] Bentley G.A., Boulot G., Karjalainen K., Mariuzza R.A. (1995). Crystal structure of the beta chain of a T cell antigen receptor. Science.

[24] Turner S.J., Doherty P.C., McCluskey J., Rossjohn J. (2006). Structural determinants of T-cell receptor bias in immunity. Nat. Rev. Immunol..

[25] Miconnet I. (2012). Probing the T-cell receptor repertoire with deep sequencing. Curr. Opin. HIV AIDS.

[26] Kent W.J. (2002). BLAT--the BLAST-like alignment tool. Genome Res..

[27] Lefranc M.P., Giudicelli V., Duroux P., Jabado-Michaloud J., Folch G., Aouinti S., Carillon E., Duvergey H., Houles A., Paysan-Lafosse T. (2015). IMGT(R), the international ImMunoGeneTics information system(R) 25 years on. Nucleic Acids Res..

[28] Shifrut E., Baruch K., Gal H., Ndifon W., Deczkowska A., Schwartz M., Friedman N. (2013). CD4(+) T Cell-Receptor Repertoire Diversity is Compromised in the Spleen but Not in the Bone Marrow of Aged Mice Due to Private and Sporadic Clonal Expansions. Front. Immunol..

[29] Crooks G.E., Hon G., Chandonia J.M., Brenner S.E. (2004). WebLogo: A sequence logo generator. Genome Res..

[30] Schneider T.D., Stephens R.M. (1990). Sequence logos: A new way to display consensus sequences. Nucleic Acids Res..

[31] Zhou S.L., Ying X.L., Han X., Sun X.X., Jin Q., Yang F. (2015). Characterization of the enterovirus 71 VP1 protein as a vaccine candidate. J. Med. Virol..

[32] Tan S., Tan X., Sun X., Lu G., Chen C.C., Yan J., Liu J., Xu W., Gao G.F. (2013). VP2 dominated CD4+ T cell responses against enterovirus 71 and cross-reactivity against coxsackievirus A16 and polioviruses in a healthy population. J. Immunol..

[33] Hoof I., Peters B., Sidney J., Pedersen L.E., Sette A., Lund O., Buus S., Nielsen M. (2009). NetMHCpan, a method for MHC class I binding prediction beyond humans. Immunogenetics.

[34] Nielsen M., Lundegaard C., Blicher T., Lamberth K., Harndahl M., Justesen S., Roder G., Peters B., Sette A., Lund O. (2007). NetMHCpan, a method for quantitative predictions of peptide binding to any HLA-A and -B locus protein of known sequence. PLoS ONE.

[35] Karosiene E., Rasmussen M., Blicher T., Lund O., Buus S., Nielsen M. (2013). NetMHCIIpan-3.0, a common pan-specific MHC class II prediction method including all three human MHC class II isotypes, HLA-DR, HLA-DP and HLA-DQ. Immunogenetics.

[36] Kozakov D., Beglov D., Bohnuud T., Mottarella S.E., Xia B., Hall D.R., Vajda S. (2013). How good is automated protein docking?. Proteins.

[37] Comeau S.R., Gatchell D.W., Vajda S., Camacho C.J. (2004). ClusPro: A fully automated algorithm for protein-protein docking. Nucleic Acids Res..

[38] Gohlke H., Hendlich M., Klebe G. (2000). Knowledge-based scoring function to predict protein-ligand interactions. J. Mol. Biol..

[39] Wallace A.C., Laskowski R.A., Thornton J.M. (1995). LIGPLOT: A program to generate schematic diagrams of protein-ligand interactions. Protein Eng..

[40] La Gruta N.L., Turner S.J. (2014). T cell mediated immunity to influenza: Mechanisms of viral control. Trends Immunol..

[41] Trautmann L., Rimbert M., Echasserieau K., Saulquin X., Neveu B., Dechanet J., Cerundolo V., Bonneville M. (2005). Selection of T cell clones expressing high-affinity public TCRs within Human cytomegalovirus-specific CD8 T cell responses. J. Immunol..

[42] Lehner P.J., Wang E.C., Moss P.A., Williams S., Platt K., Friedman S.M., Bell J.I., Borysiewicz L.K. (1995). Human HLA-A0201-restricted cytotoxic T lymphocyte recognition of influenza A is dominated by T cells bearing the V beta 17 gene segment. J. Exp. Med..

[43] Argaet V.P., Schmidt C.W., Burrows S.R., Silins S.L., Kurilla M.G., Doolan D.L., Suhrbier A., Moss D.J., Kieff E., Sculley T.B. (1994). Dominant selection of an invariant T cell antigen receptor in response to persistent infection by Epstein-Barr virus. J. Exp. Med..

[44] Kapasi Z.F., Murali-Krishna K., McRae M.L., Ahmed R. (2002). Defective generation but normal maintenance of memory T cells in old mice. Eur. J. Immunol..

[45] Dong C., Wang J., Liu L., Zhao H., Shi H., Zhang Y., Jiang L., Li Q. (2010). Optimized development of a candidate strain of inactivated EV71 vaccine and analysis of its immunogenicity in rhesus monkeys. Hum. Vaccines.

[46] Ong K.C., Devi S., Cardosa M.J., Wong K.T. (2010). Formaldehyde-inactivated whole-virus vaccine protects a murine model of enterovirus 71 encephalomyelitis against disease. J. Virol..

[47] Foo D.G., Alonso S., Phoon M.C., Ramachandran N.P., Chow V.T., Poh C.L. (2007). Identification of neutralizing linear epitopes from the VP1 capsid protein of Enterovirus 71 using synthetic peptides. Virus Res..

[48] Tung W.S., Bakar S.A., Sekawi Z., Rosli R. (2007). DNA vaccine constructs against enterovirus 71 elicit immune response in mice. Genet. Vaccines Ther..

[49] Chung Y.C., Ho M.S., Wu J.C., Chen W.J., Huang J.H., Chou S.T., Hu Y.C. (2008). Immunization with virus-like particles of enterovirus 71 elicits potent immune responses and protects mice against lethal challenge. Vaccine.

[50] Liang Z., Wang J. (2014). EV71 vaccine, an invaluable gift for children. Clin. Transl. Immunol..

[51] Mao Q., Dong C., Li X., Gao Q., Guo Z., Yao X., Wang Y., Gao F., Li F., Xu M. (2012). Comparative analysis of the immunogenicity and protective effects of inactivated EV71 vaccines in mice. PLoS ONE.

[52] Liu L., Mo Z., Liang Z., Zhang Y., Li R., Ong K.C., Wong K.T., Yang E., Che Y., Wang J. (2015). Immunity and clinical efficacy of an inactivated enterovirus 71 vaccine in healthy Chinese children: A report of further observations. BMC Med..

[53] Wu C.Y., Lin Y.W., Kuo C.H., Liu W.H., Tai H.F., Pan C.H., Chen Y.T., Hsiao P.W., Chan C.H., Chang C.C. (2015). Inactivated Enterovirus 71 Vaccine Produced by 200-L Scale Serum-Free Microcarrier Bioreactor System Provides Cross-Protective Efficacy in Human SCARB2 Transgenic Mouse. PLoS ONE.

[54] Chen M., Ju Y., Chen M., Xie Z., Zhou K., Tan Y., Mo J. (2017). Epidemiological and genetic characteristics of EV71 in hand, foot, and mouth disease in Guangxi, southern China, from 2010 to 2015. PLoS ONE.

[55] Noisumdaeng P., Sangsiriwut K., Prasertsopon J., Klinmalai C., Payungporn S., Mungaomklang A., Chokephaibulkit K., Buathong R., Thitithanyanont A., Puthavathana P. (2018). Complete genome analysis demonstrates multiple introductions of enterovirus 71 and coxsackievirus A16 recombinant strains into Thailand during the past decade. Emerg Microbes Infect.

[56] Esfandiarei M., McManus B.M. (2008). Molecular biology and pathogenesis of viral myocarditis. Annu. Rev. Pathol..

[57] Karussis D. (2014). The diagnosis of multiple sclerosis and the various related demyelinating syndromes: A critical review. J. Autoimmun..

[58] Polman C.H., O’Connor P.W., Havrdova E., Hutchinson M., Kappos L., Miller D.H., Phillips J.T., Lublin F.D., Giovannoni G., Wajgt A. (2006). A randomized, placebo-controlled trial of natalizumab for relapsing multiple sclerosis. N. Engl. J. Med..

[59] Fernandez-Malave E., Stark-Aroeira L. (2011). A natural anti-T-cell receptor monoclonal antibody protects against experimental autoimmune encephalomyelitis. J. Neuroimmunol..

[60] Wu H., Walters G., Knight J.F., Alexander S.I. (2003). DNA vaccination against specific pathogenic TCRs reduces proteinuria in active Heymann nephritis by inducing specific autoantibodies. J. Immunol..

[61] Yang C.H., Liang C.T., Jiang S.T., Chen K.H., Yang C.C., Cheng M.L., Ho H.Y. (2019). A Novel Murine Model Expressing a Chimeric mSCARB2/hSCARB2 Receptor Is Highly Susceptible to Oral Infection with Clinical Isolates of Enterovirus 71. J. Virol..

[62] Chang C.S., Liao C.C., Liou A.T., Chang Y.S., Chang Y.T., Tzeng B.H., Chen C.C., Shih C. (2019). Enterovirus 71 targets the cardiopulmonary system in a robust oral infection mouse model. Sci. Rep..

[63] Shih C., Liao C.C., Chang Y.S., Wu S.Y., Chang C.S., Liou A.T. (2018). Immunocompetent and Immunodeficient Mouse Models for Enterovirus 71 Pathogenesis and Therapy. Viruses.

[64] Wang Y.F., Chou C.T., Lei H.Y., Liu C.C., Wang S.M., Yan J.J., Su I.J., Wang J.R., Yeh T.M., Chen S.H. (2004). A mouse-adapted enterovirus 71 strain causes neurological disease in mice after oral infection. J. Virol..

[65] Yousfi Monod M., Giudicelli V., Chaume D., Lefranc M.P. (2004). IMGT/JunctionAnalysis: The first tool for the analysis of the immunoglobulin and T cell receptor complex V-J and V-D-J JUNCTIONs. Bioinformatics.

[66] Lang P.T., Brozell S.R., Mukherjee S., Pettersen E.F., Meng E.C., Thomas V., Rizzo R.C., Case D.A., James T.L., Kuntz I.D. (2009). DOCK 6: Combining techniques to model RNA-small molecule complexes. RNA.

[67] Sali A., Blundell T.L. (1993). Comparative protein modelling by satisfaction of spatial restraints. J. Mol. Biol..

[68] Chen M.H., Sandberg D.J., Babu K.R., Bubis J., Surya A., Ramos L.S., Zapata H.J., Galan J.F., Sandberg M.N., Birge R.R. (2011). Conserved residues in the extracellular loops of short-wavelength cone visual pigments. Biochemistry.

[69] Biasini M., Bienert S., Waterhouse A., Arnold K., Studer G., Schmidt T., Kiefer F., Cassarino T.G., Bertoni M., Bordoli L. (2014). SWISS-MODEL: Modelling protein tertiary and quaternary structure using evolutionary information. Nucleic Acids Res..

[70] Guex N., Peitsch M.C., Schwede T. (2009). Automated comparative protein structure modeling with SWISS-MODEL and Swiss-PdbViewer: A historical perspective. Electrophoresis.

[71] Kiefer F., Arnold K., Kunzli M., Bordoli L., Schwede T. (2009). The SWISS-MODEL Repository and associated resources. Nucleic Acids Res..

[72] Chen V.B., Arendall W.B., Headd J.J., Keedy D.A., Immormino R.M., Kapral G.J., Murray L.W., Richardson J.S., Richardson D.C. (2010). MolProbity: All-atom structure validation for macromolecular crystallography. Acta Crystallogr. Sect. Dbiol. Crystallogr..

[73] Davis I.W., Leaver-Fay A., Chen V.B., Block J.N., Kapral G.J., Wang X., Murray L.W., Arendall W.B., Snoeyink J., Richardson J.S. (2007). MolProbity: All-atom contacts and structure validation for proteins and nucleic acids. Nucleic Acids Res..

[74] Phillips J.C., Braun R., Wang W., Gumbart J., Tajkhorshid E., Villa E., Chipot C., Skeel R.D., Kale L., Schulten K. (2005). Scalable molecular dynamics with NAMD. J. Comput. Chem..

[75] Humphrey W., Dalke A., Schulten K. (1996). VMD: Visual molecular dynamics. J. Mol. Graph..

[76] Pettersen E.F., Goddard T.D., Huang C.C., Couch G.S., Greenblatt D.M., Meng E.C., Ferrin T.E. (2004). UCSF Chimera–a visualization system for exploratory research and analysis. J. Comput. Chem..

