# Supplementary material for: Enterovirus 71 Infection Shapes Host T Cell Receptor Repertoire and Presumably Expands VP1-Specific TCRβ CDR3 Cluster
Source: Pathogens. 2020 Feb 14;9(2):121. doi: 10.3390/pathogens9020121 (PMC7169398; doi:10.3390/pathogens9020121)
Supplement: Supplementary file 1 [file pathogens-09-00121-s001.pdf]

Enterovirus 71 Infection Shapes Host T Cell Receptor Repertoire and Presumably Expands VP1-Specific TCRβ CDR3 Cluster

Yu-Wen Liao, Bing-Ching Ho, Min-Hsuan Chen, Sung-Liang Yu

Supplementary Material

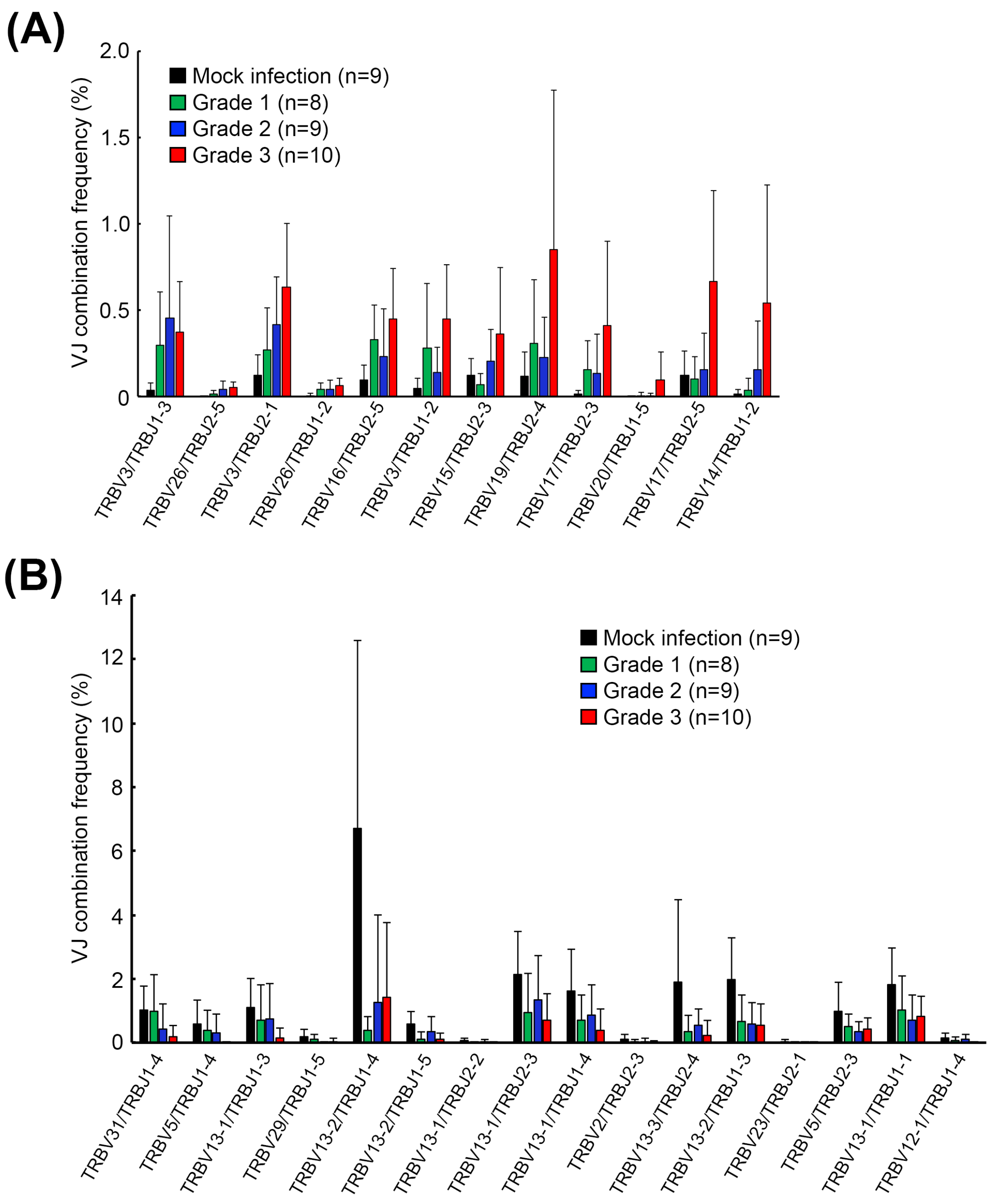

Supplementary Figure 1. Differential usages of VJ combinations in brainstems of mEV71-infected mice, referred to Figure 2

The VJ combination usages in the brainstem of mice with different clinical symptom severities. The VJ combination usages with significant *p* value of trend test in mEV71 infection are illustrated. 12 VJ combination usages were significantly increased (A) and of 16 were decreased (B) with grading in mEV71 infection. *p* value of trend test <0.05.

(A)

EV71 VP1 peptide: FTYMRFDAEFTF (691 a.a.-702 a.a.)

EV71/AF304457.1	F	T	Y	M	R	F	D	A	E	F	T	F
mEV71	F	T	Y	M	R	F	D	A	E	F	T	F

(B)

EV71 VP1 peptide: LAWQTATNPSVF (734 a.a.-745 a.a.)

EV71/AF304457.1	L	A	W	Q	T	A	T	N	P	S	V	F
mEV71	L	A	W	Q	T	A	T	N	P	S	V	F

(C)

EV71 VP1 peptide: IYMRMKHVRAWI (816 a.a.-827 a.a.)

EV71/AF304457.1	I	Y	M	R	M	K	H	V	R	A	W	I
mEV71	I	Y	M	R	M	K	H	V	R	A	W	I

(D)

EV71 VP1 peptide: SFFSRAGLVGEI (647 a.a.-658 a.a.)

EV71/AF304457.1	S	F	F	S	R	A	G	L	V	G	E	I
mEV71	S	F	F	S	R	A	G	L	V	G	E	I

Supplementary Figure 2. Amino acid sequences of cleaved mEV71 VP1 peptides

The amino acid sequences of cleaved mEV71 VP1 peptides were identical to Enterovirus 71 strain Tainan/5746/9 (AF304457.1). mEV71 were applied to viral RNA extraction followed by RT-PCR. The PCR fragments were then sequenced and aligned to Enterovirus 71 strain Tainan/5746/9 which was used for viral peptide cleavage prediction and ligand-binding prediction in this study. Alignments of EV71 VP1 peptides FTYMRFDAEFTF (A), LAWQTATNPSVF (B), IYMRMKHVRAWI (C), and SFFSRAGLVGEI (D), were indicated.

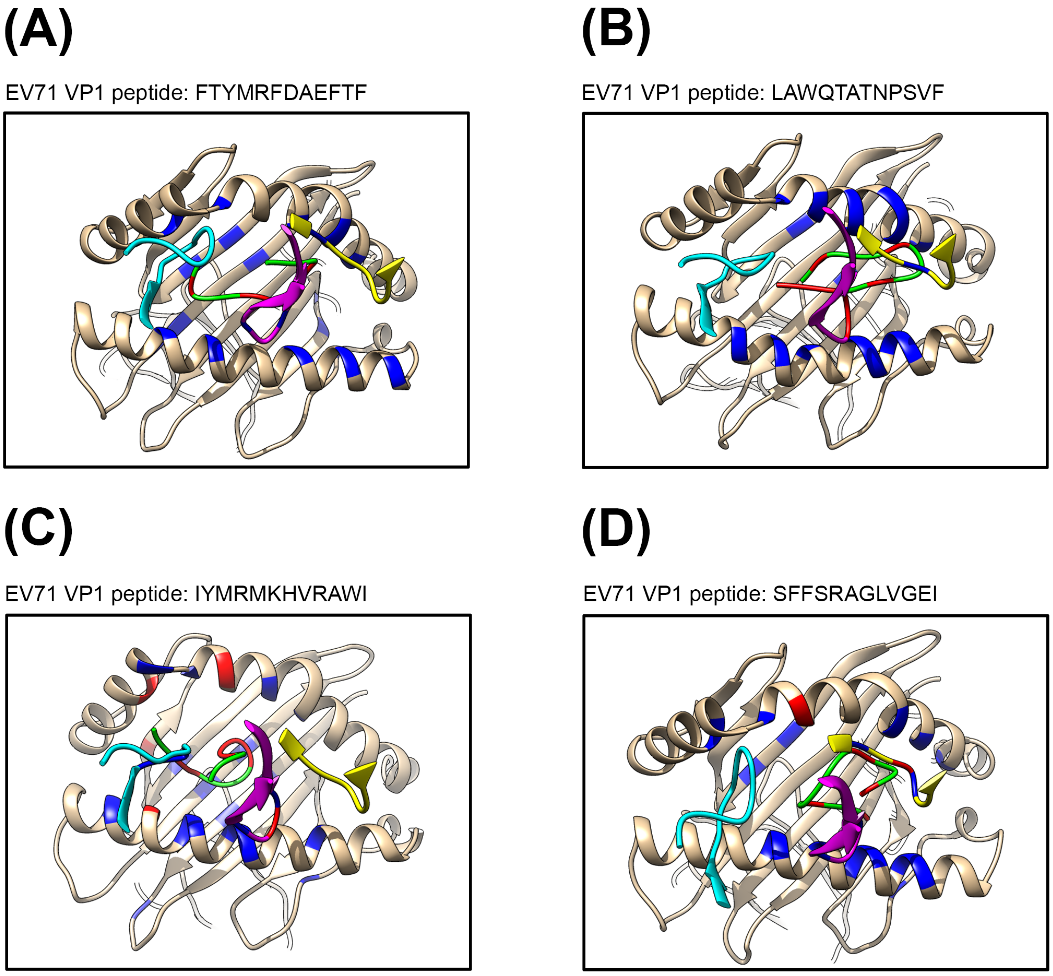

Supplementary Figure 3. The binding complex between TCRβ CDR3, EV71 VP1 peptide and MHC class I, referred to Figure 4

The binding complexes composed by TCRβ CDR3 CASSLGANSDYTF, MHC class I molecule and cleaved EV71 VP1 peptide are illustrated. The binding complexes composed with cleaved EV71 VP1 peptide FTYMRFDAEFTF (A), LAWQTATNPSVF (B), IYMRMKHVRAWI (C), and SFFSRAGLVGEI (D). The binding groove in MHCI with the loops that are in proximity of the viral peptide is presented in ribbons. The viral peptide is presented in green; residues that have hydrophobic interactions towards viral peptide in navy blue; those that have hydrogen binding in red; the CDR3 loop in the TCRβ in light blue.

(A)

EV71 VP1 peptide sequence: FTYMRFDAEFTF

CDR3 sequence: CASSLGANSDYTF

CDR3 sequence: CANSGLSSDAYTF

CDR3 sequence: CGAYLANSSSDTF

CDR3 sequence: CGSNALYSTDASF

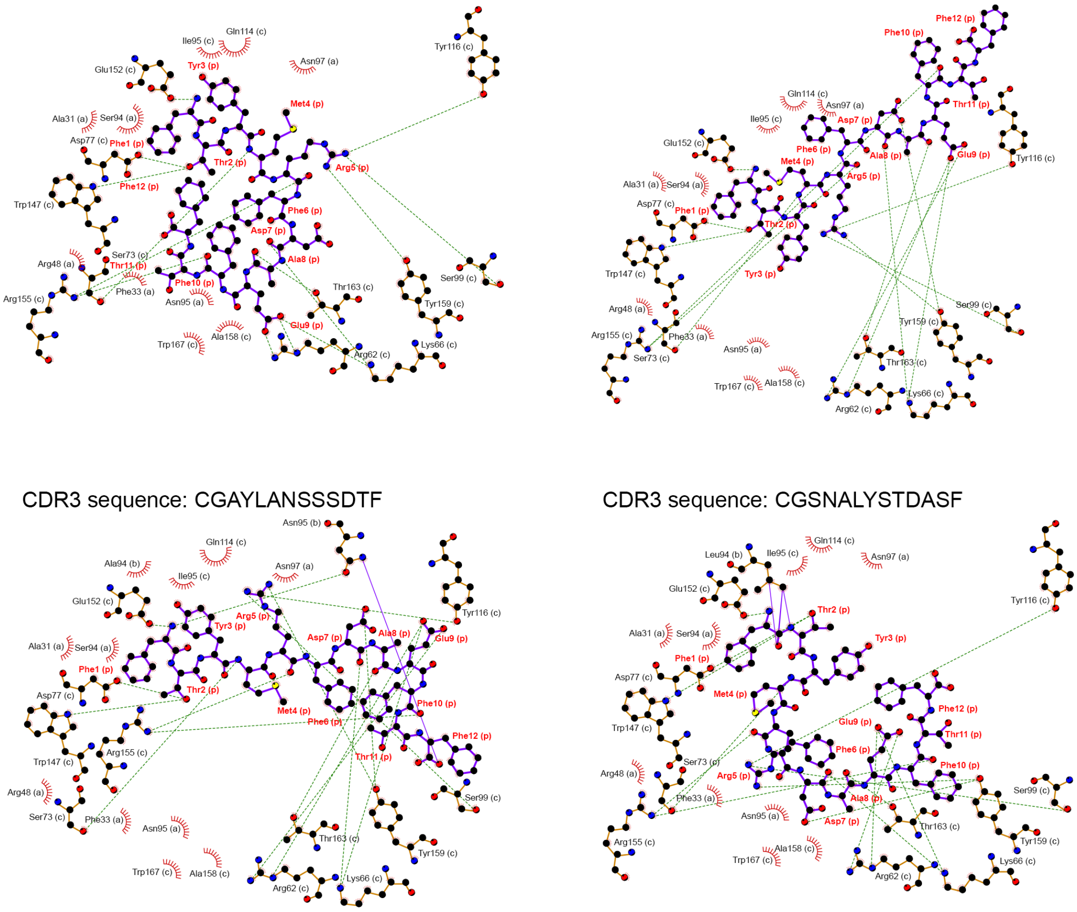

(B)

EV71 VP1 peptide sequence: LAWQTATNPSVF

CDR3 sequence: CASSLGANSDYTF

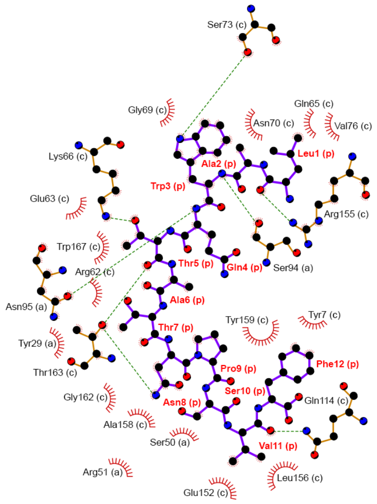

CDR3 sequence: CLATGNSAYDSSF

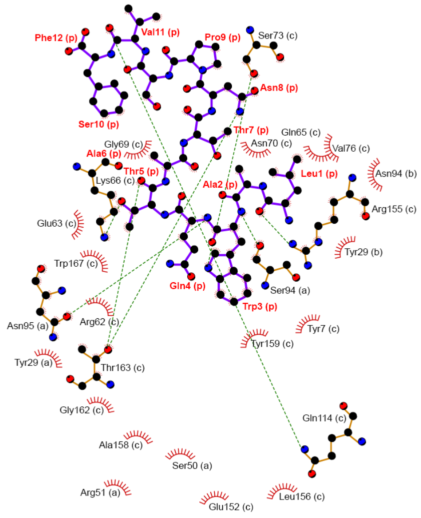

CDR3 sequence: CLSYATASNGSDF

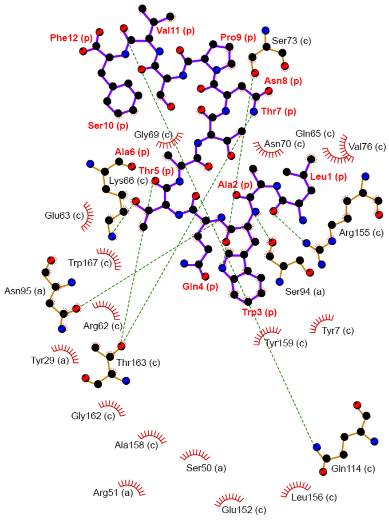

CDR3 sequence: CTSNGAASSDLYF

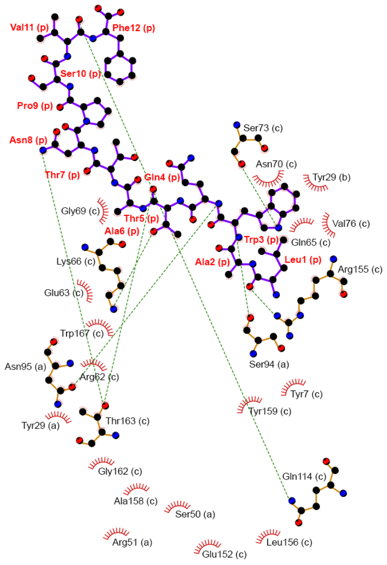

(C)

EV71 VP1 peptide sequence: IYMRMKHVRAWI

CDR3 sequence: CASSLGANSDYTF

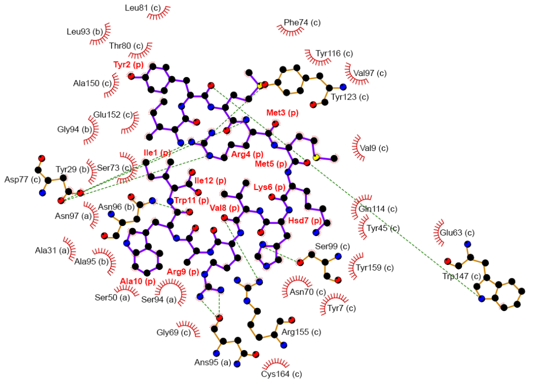

CDR3 sequence: CAALYTDSSGSNF

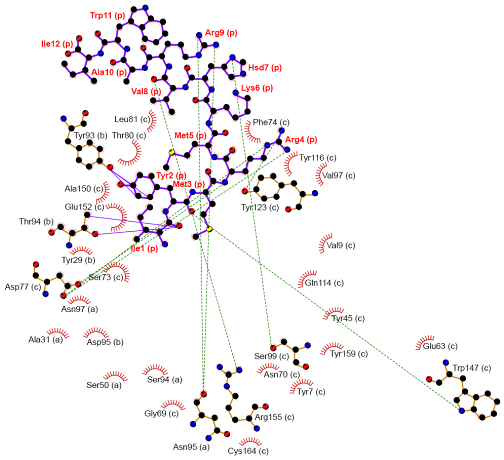

CDR3 sequence: CLTAAGNDYSSSF

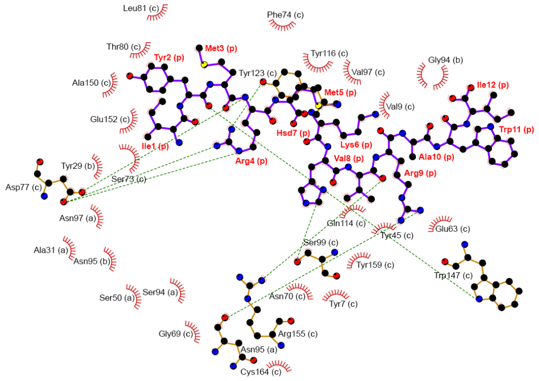

CDR3 sequence: CSTSNDSAYLGAF

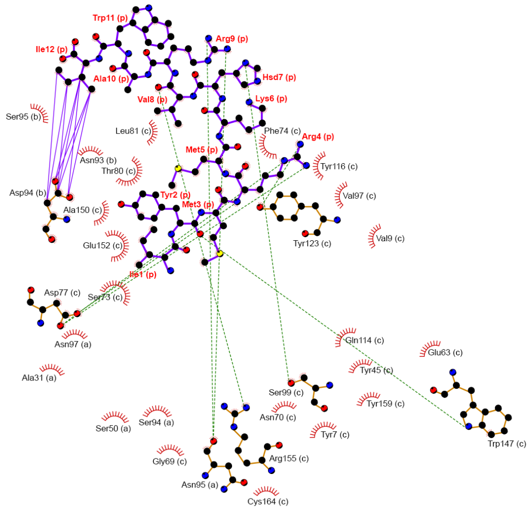

(D)

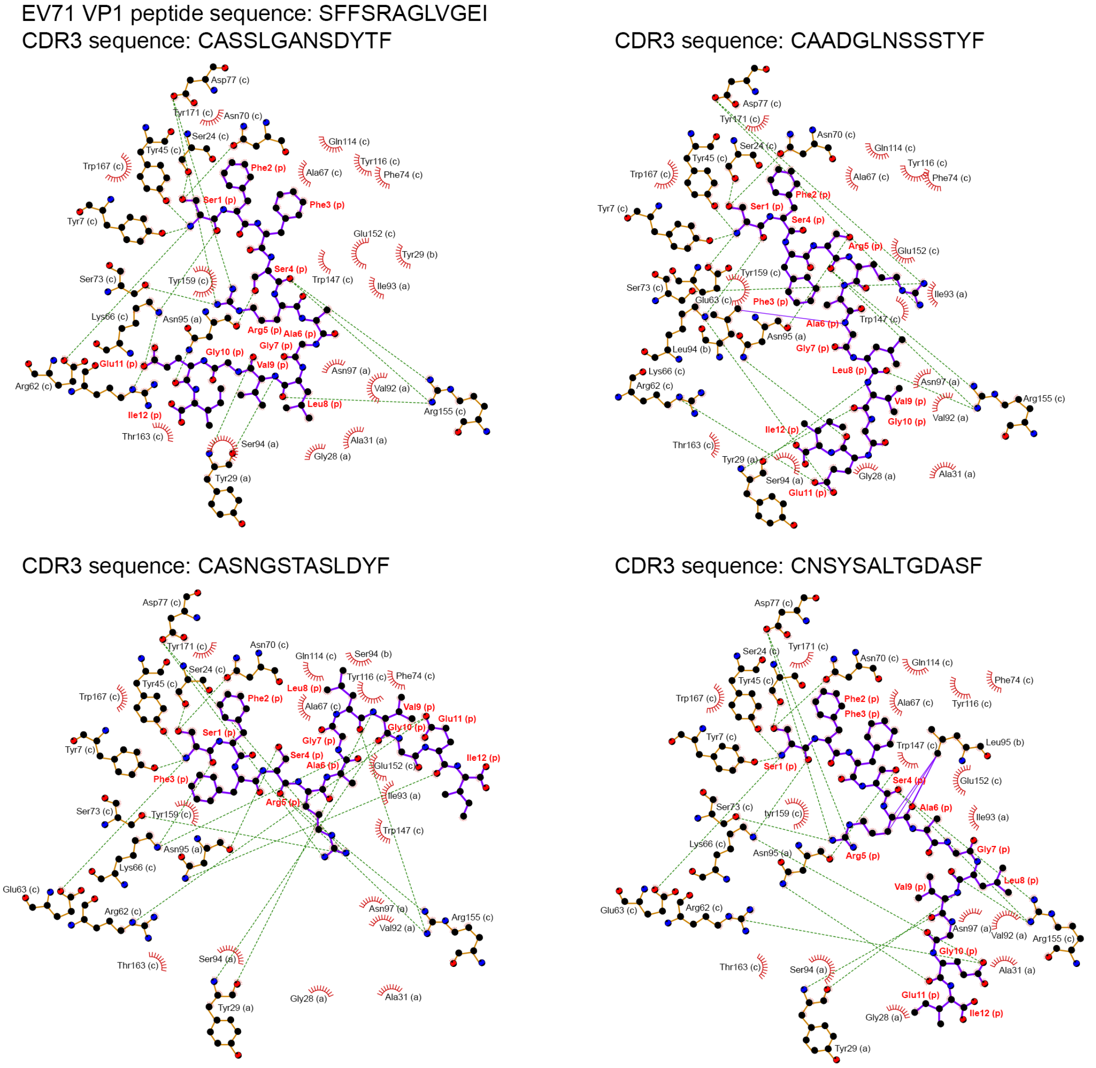

Supplementary Figure 4. The binding complex between scrambled TCRβ CDR3, cleaved EV71 VP1 peptide and MHC class I, referred to Figure 4

The binding complexes composed by scrambled TCRβ CDR3 CASSLGANSDYTF, MHC class I molecule and cleaved VP1 peptide are illustrated, where the spatial orientation between interacting side chains was considered. The binding complexes composed with cleaved EV71 VP1 peptides FTYMRFDAEFTF (A), LAWQTATNPSVF (B), IYMRMKHVRAWI (C), and SFFSRAGLVGEI (D). The green dash line represents hydrogen binding; the red spike represents hydrophobic interactions; the alphabet in parenthesis represents the chain ID in protein complex; the ball and stick in black and purple represent the cleaved EV71 VP1 peptide.

Supplementary Table 1 The statistics of mapped sequencing reads in each severity-classified group

	**Severity grade**			
	**Mock infection**	**Grade 1**	**Grade 2**	**Grade 3**
No. of mice	9	8	9	10
Average mapped reads	1,208,797	1,227,789	995,237	1,026,699
Range of mapped reads	831,507-1,680,923	695,253-1,932,487	347,050-1,410,174	190,526-1,776,472

Supplementary Table 2 Potential cleaved EV71 VP1 epitopes presented by MHCI according to mouse alleles

Cleaved EV71 VP1 peptide	H-2-Db[a]	H-2-Dd	H-2-Kb	H-2-Kd	H-2-Kk	H-2-Ld	Ave. 1-log50k	NB
FTYMRFDAEFTF	0.27	0.46	0.65	0.26	0.35	0.26	0.37	3
LAWQTATNPSVF	0.33	0.28	0.39	0.32	0.40	0.45	0.36	3
IYMRMKHVRAWI	0.19	0.24	0.41	0.88	0.45	0.26	0.41	2
SFFSRAGLVGEI	0.13	0.18	0.47	0.65	0.33	0.18	0.32	2
TYMRFDAEFTFV	0.22	0.17	0.37	0.65	0.30	0.19	0.32	1
QMRRKVELFTYM	0.19	0.21	0.59	0.35	0.28	0.22	0.31	1
IPRPMRNQNYLF	0.07	0.14	0.30	0.13	0.36	0.61	0.27	1
KYPLVIRIYMRM	0.07	0.24	0.38	0.47	0.11	0.13	0.23	1
YGACPNNMMGTF	0.17	0.28	0.25	0.27	0.19	0.20	0.23	1
TGYAQMRRKVEL	0.12	0.22	0.50	0.21	0.15	0.15	0.23	1
WIPRPMRNQNYL	0.23	0.32	0.36	0.21	0.11	0.10	0.22	1
MKHVRAWIPRPM	0.07	0.07	0.29	0.12	0.39	0.34	0.21	1
SIKPTGASRTAI	0.10	0.10	0.28	0.44	0.24	0.12	0.21	1
CTPTGEVVPQLL	0.16	0.46	0.30	0.19	0.10	0.06	0.21	1
VGTSKSKYPLVI	0.11	0.31	0.36	0.20	0.17	0.10	0.21	1
VPFMSPASAYQW	0.06	0.07	0.18	0.07	0.41	0.44	0.20	1
GASSNASDESMI	0.30	0.14	0.16	0.35	0.19	0.09	0.20	1
TTLDSFFSRAGL	0.14	0.16	0.45	0.17	0.17	0.11	0.20	1

SAYQWFYDGYPT	0.20	0.08	0.47	0.11	0.21	0.12	0.20	1
KSKYPLVIRIYM	0.12	0.20	0.44	0.17	0.11	0.12	0.19	1
RIYMRMKHVRAW	0.07	0.12	0.44	0.21	0.17	0.12	0.19	1
LSDPPAQVSVPF	0.09	0.31	0.34	0.09	0.15	0.12	0.19	1
RPMRNQNYLFKA	0.14	0.04	0.14	0.15	0.19	0.38	0.17	1
LVIRIYMRMKHV	0.06	0.05	0.47	0.10	0.15	0.06	0.15	1
APKPDSRESLAW	0.03	0.03	0.07	0.04	0.20	0.30	0.11	1
LPLEGTTNPNGY	0.04	0.03	0.09	0.04	0.19	0.28	0.11	1

[a]H-2-Db, H-2-Dd, H-2-Kb, H-2-Kd, H-2-Kk, and H-2-Ld are mouse MHCI molecules. 1-log50K indicates the predicted binding affinity of epitope binding to specific MHCI molecule and is presented in log-scale. NB: number of binding.

Supplementary Table 3 Potential cleaved EV71 VP1 epitopes presented by MHCII according to mouse alleles

Cleaved EV71 VP1 peptide	H-2-IAb[a]	H-2-IAd	Ave. 1-log50k	NB
VSRALTRALPAPTGQ	0.43	0.38	0.40	2
AQVSVPFMSPASAYQ	0.45	0.26	0.36	1
VPALQAAEIGASSNA	0.42	0.30	0.36	1
ESLAWQTATNPSVFV	0.45	0.26	0.35	1
QLLQYMFVPPGAPKP	0.43	0.19	0.31	1
IRIYMRMKHVRAWIP	0.22	0.36	0.29	1

[a]H-2-IAb or H-2-IAd are mouse MHCII molecules. 1-log50K indicates the predicted binding affinity of epitope binding to specific MHCII molecule and is presented in log-scale. NB: number of binding.

Supplementary Table 4 Potential cleaved EV71 VP2 epitopes presented by MHCI according to mouse alleles

Cleaved EV71 VP2 peptide	H-2-Db[a]	H-2-Dd	H-2-Kb	H-2-Kd	H-2-Kk	H-2-Ld	Ave. 1-log50k	NB
CGYSDRVAQLTI	0.19	0.18	0.31	0.34	0.23	0.10	0.23	2
CNASKFHQGALL	0.05	0.20	0.44	0.16	0.16	0.11	0.19	2
DSALNHCNFGLL	0.25	0.19	0.33	0.12	0.14	0.11	0.19	2
SALNHCNFGLLV	0.32	0.10	0.47	0.15	0.17	0.16	0.23	2
STITTQEAANII	0.28	0.11	0.23	0.30	0.18	0.07	0.19	1
KPTRPDVSVNRF	0.03	0.06	0.06	0.08	0.09	0.29	0.10	1
RPDVSVNRFYTL	0.09	0.13	0.11	0.11	0.15	0.44	0.17	1
SVNRFYTLDTKL	0.15	0.12	0.47	0.20	0.10	0.10	0.19	1
WEKSSKGWYWKF	0.03	0.03	0.06	0.08	0.47	0.08	0.12	1
TETGVFGQNAQF	0.02	0.05	0.06	0.07	0.40	0.04	0.11	1
GVFGQNAQFHYL	0.11	0.11	0.44	0.14	0.08	0.09	0.16	1
NAQFHYLYRSGF	0.07	0.16	0.34	0.14	0.18	0.23	0.19	1
HYLYRSGFCIHV	0.07	0.04	0.30	0.32	0.11	0.11	0.16	1
KFHQGALLVAVL	0.13	0.06	0.19	0.41	0.09	0.13	0.17	1
GALLVAVLPEYV	0.26	0.05	0.15	0.08	0.08	0.06	0.12	1
ISQLTVCPHQWI	0.19	0.19	0.27	0.22	0.15	0.08	0.18	1
VPYINALPFDSA	0.14	0.03	0.10	0.06	0.16	0.29	0.13	1
PYINALPFDSAL	0.06	0.05	0.17	0.43	0.04	0.07	0.14	1

LPFDSALNHCNF	0.06	0.08	0.14	0.08	0.22	0.50	0.18	1
NHCNFGLLVVPI	0.07	0.08	0.20	0.24	0.42	0.12	0.19	1
SPLDYDQGATPV	0.09	0.02	0.08	0.08	0.20	0.24	0.12	1
ITITLAPMCSEF	0.15	0.25	0.41	0.24	0.11	0.13	0.21	1

[a]H-2-Db, H-2-Dd, H-2-Kb, H-2-Kd, H-2-Kk, and H-2-Ld are mouse MHCI molecules. 1-log50K indicates the predicted binding affinity of epitope binding to specific MHCI molecule and is presented in log-scale. NB: number of binding.

Supplementary Table 5 Potential cleaved EV71 VP2 epitopes presented by MHCII according to mouse alleles

Cleaved EV71 VP2 peptide	H-2-IAb[a]	H-2-IAd	Ave. 1-log50k	NB
PLDYDQGATPVIPIT	0.40	0.16	0.28	0
HPYVLDAGIPISQLT	0.38	0.29	0.34	0
IVPYINALPFDSALN	0.38	0.31	0.35	0
NASKFHQGALLVAVL	0.34	0.35	0.35	0
PMCSEFAGLRQAVTQ	0.32	0.28	0.30	0
CNASKFHQGALLVAV	0.32	0.32	0.32	0
YINALPFDSALNHCN	0.31	0.24	0.28	0
APMCSEFAGLRQAVT	0.31	0.26	0.28	0
SKFHQGALLVAVLPE	0.31	0.32	0.31	0
FELQHPYVLDAGIPI	0.30	0.22	0.26	0
PISPLDYDQGATPVI	0.29	0.12	0.20	0
LPEYVIGTVAGGTGT	0.29	0.13	0.21	0
ALPFDSALNHCNFGL	0.29	0.16	0.22	0
QCNASKFHQGALLVA	0.29	0.27	0.28	0
PEYVIGTVAGGTGTE	0.28	0.11	0.20	0
NSTITTQEAANIIVG	0.28	0.31	0.30	0
QFHYLYRSGFCIHVQ	0.28	0.17	0.22	0
INALPFDSALNHCNF	0.28	0.18	0.23	0
NALPFDSALNHCNFG	0.28	0.15	0.21	0
GNSTITTQEAANIIV	0.28	0.30	0.29	0
VLPEYVIGTVAGGTG	0.28	0.13	0.20	0
KFHQGALLVAVLPEY	0.27	0.29	0.28	0
FHYLYRSGFCIHVQC	0.27	0.16	0.22	0
HYLYRSGFCIHVQCN	0.27	0.16	0.22	0
STITTQEAANIIVGY	0.27	0.30	0.29	0
VLDAGIPISQLTVCP	0.27	0.22	0.25	0
AQFHYLYRSGFCIHV	0.27	0.16	0.22	0
GFELQHPYVLDAGIP	0.27	0.20	0.23	0
LAPMCSEFAGLRQAV	0.27	0.24	0.25	0
AVLPEYVIGTVAGGT	0.27	0.14	0.20	0
ADGFELQHPYVLDAG	0.26	0.19	0.22	0

LPFDSALNHCNFGLL	0.26	0.14	0.20	0
GADGFELQHPYVLDA	0.26	0.18	0.22	0
SKGWYWKFPDVLTET	0.26	0.15	0.20	0
EYVIGTVAGGTGTED	0.25	0.09	0.17	0
HPPYKQTQPGADGFE	0.25	0.08	0.17	0
FHQGALLVAVLPEYV	0.25	0.25	0.25	0
IGNSTITTQEAANII	0.25	0.28	0.27	0
KGWYWKFPDVLTETG	0.25	0.15	0.20	0
SHPPYKQTQPGADGF	0.25	0.08	0.17	0
IPITITLAPMCSEFA	0.25	0.28	0.26	0
DGFELQHPYVLDAGI	0.25	0.19	0.22	0
YLYRSGFCIHVQCNA	0.25	0.16	0.20	0
RVAQLTIGNSTITTQ	0.25	0.21	0.23	0
TITTQEAANIIVGYG	0.24	0.27	0.26	0
VIPITITLAPMCSEF	0.24	0.27	0.26	0
GWYWKFPDVLTETGV	0.24	0.16	0.20	0
SSKGWYWKFPDVLTE	0.24	0.14	0.19	0
PPYKQTQPGADGFEL	0.24	0.08	0.16	0
DRVAQLTIGNSTITT	0.24	0.22	0.23	0
PITITLAPMCSEFAG	0.24	0.27	0.25	0
YVIGTVAGGTGTEDS	0.23	0.08	0.15	0
CNFGLLVVPISPLDY	0.23	0.22	0.23	0
VAVLPEYVIGTVAGG	0.23	0.14	0.19	0
GALLVAVLPEYVIGT	0.23	0.23	0.23	0
PVIPITITLAPMCSE	0.23	0.26	0.24	0
VAQLTIGNSTITTQE	0.23	0.18	0.20	0
HQGALLVAVLPEYVI	0.23	0.23	0.23	0
QGALLVAVLPEYVIG	0.23	0.23	0.23	0
NFGLLVVPISPLDYD	0.23	0.22	0.23	0
DQGATPVIPITITLA	0.23	0.17	0.20	0
TLAPMCSEFAGLRQA	0.23	0.21	0.22	0
ACGYSDRVAQLTIGN	0.23	0.21	0.22	0
CATIIVPYINALPFD	0.23	0.18	0.20	0
SDRVAQLTIGNSTIT	0.22	0.23	0.23	0

PFDSALNHCNFGLLV	0.22	0.12	0.17	0
TPVIPITITLAPMCS	0.22	0.24	0.23	0
EACGYSDRVAQLTIG	0.22	0.20	0.21	0
LYRSGFCIHVQCNAS	0.22	0.16	0.19	0
PGADGFELQHPYVLD	0.22	0.14	0.18	0
TIGNSTITTQEAANI	0.22	0.25	0.24	0
ITITLAPMCSEFAGL	0.22	0.24	0.23	0
PYKQTQPGADGFELQ	0.22	0.08	0.15	0
QPGADGFELQHPYVL	0.22	0.14	0.18	0
TITLAPMCSEFAGLR	0.22	0.22	0.22	0
FGLLVVPISPLDYDQ	0.22	0.22	0.22	0
AQLTIGNSTITTQEA	0.22	0.18	0.20	0
TETGVFGQNAQFHYL	0.22	0.17	0.20	0
WYWKFPDVLTETGVF	0.22	0.15	0.19	0
HCNFGLLVVPISPLD	0.22	0.21	0.21	0
ETGVFGQNAQFHYLY	0.22	0.19	0.20	0
LDAGIPISQLTVCPH	0.22	0.21	0.21	0
LLVAVLPEYVIGTVA	0.21	0.20	0.20	0
QLTIGNSTITTQEAA	0.21	0.19	0.20	0
AEACGYSDRVAQLTI	0.21	0.18	0.20	0
ALLVAVLPEYVIGTV	0.21	0.21	0.21	0
GYSDRVAQLTIGNST	0.21	0.23	0.22	0
LVAVLPEYVIGTVAG	0.21	0.17	0.19	0
CGYSDRVAQLTIGNS	0.21	0.23	0.22	0
DSHPPYKQTQPGADG	0.21	0.06	0.13	0
KSSKGWYWKFPDVLT	0.21	0.11	0.16	0
YSDRVAQLTIGNSTI	0.21	0.24	0.22	0
NCATIIVPYINALPF	0.21	0.18	0.19	0
ITTQEAANIIVGYGE	0.21	0.23	0.22	0
NHCNFGLLVVPISPL	0.21	0.21	0.21	0
LTETGVFGQNAQFHY	0.21	0.16	0.18	0
YRSGFCIHVQCNASK	0.20	0.16	0.18	0
LTIGNSTITTQEAAN	0.20	0.19	0.20	0
TGVFGQNAQFHYLYR	0.20	0.18	0.19	0

ITLAPMCSEFAGLRQ	0.20	0.20	0.20	0
WPSYCSDSDATAVDK	0.20	0.11	0.15	0
QGATPVIPITITLAP	0.20	0.16	0.18	0
ATPVIPITITLAPMC	0.19	0.22	0.21	0
VQCNASKFHQGALLV	0.19	0.21	0.20	0
SAEACGYSDRVAQLT	0.19	0.15	0.17	0
VIGTVAGGTGTEDSH	0.19	0.06	0.13	0
NNCATIIVPYINALP	0.19	0.17	0.18	0
VLTETGVFGQNAQFH	0.19	0.16	0.18	0
EDSHPPYKQTQPGAD	0.19	0.05	0.12	0
GLLVVPISPLDYDQG	0.19	0.19	0.19	0
EWPSYCSDSDATAVD	0.19	0.10	0.14	0
QWINLRTNNCATIIV	0.18	0.19	0.19	0
GATPVIPITITLAPM	0.18	0.19	0.19	0
HQWINLRTNNCATII	0.18	0.18	0.18	0
GEWPSYCSDSDATAV	0.18	0.09	0.14	0
PSYCSDSDATAVDKP	0.18	0.10	0.14	0
GVFGQNAQFHYLYRS	0.18	0.18	0.18	0
GFCIHVQCNASKFHQ	0.18	0.19	0.18	0
FDSALNHCNFGLLVV	0.18	0.12	0.15	0
TNNCATIIVPYINAL	0.18	0.17	0.17	0
WINLRTNNCATIIVP	0.17	0.18	0.18	0
SGFCIHVQCNASKFH	0.17	0.18	0.18	0
RSGFCIHVQCNASKF	0.17	0.17	0.17	0
NAQFHYLYRSGFCIH	0.17	0.15	0.16	0
IIVGYGEWPSYCSDS	0.17	0.09	0.13	0
EKSSKGWYWKFPDVL	0.17	0.08	0.13	0
PHQWINLRTNNCATI	0.17	0.17	0.17	0
FCIHVQCNASKFHQG	0.17	0.18	0.18	0
LNHCNFGLLVVPISP	0.17	0.17	0.17	0
HVQCNASKFHQGALL	0.17	0.16	0.16	0
DVLTETGVFGQNAQF	0.17	0.14	0.15	0
ANIIVGYGEWPSYCS	0.17	0.09	0.13	0
YKQTQPGADGFELQH	0.17	0.08	0.12	0

INLRTNNCATIIVPY	0.17	0.18	0.17	0
SYCSDSDATAVDKPT	0.17	0.10	0.13	0
NIIVGYGEWPSYCSD	0.17	0.09	0.13	0
IVGYGEWPSYCSDSD	0.17	0.08	0.12	0
CIHVQCNASKFHQGA	0.16	0.17	0.16	0
YGEWPSYCSDSDATA	0.16	0.08	0.12	0
IGTVAGGTGTEDSHP	0.16	0.05	0.11	0
TTQEAANIIVGYGEW	0.16	0.16	0.16	0
QNAQFHYLYRSGFCI	0.16	0.16	0.16	0
DAGIPISQLTVCPHQ	0.16	0.18	0.17	0
YWKFPDVLTETGVFG	0.16	0.14	0.15	0
IHVQCNASKFHQGAL	0.16	0.15	0.15	0
VFGQNAQFHYLYRSG	0.16	0.18	0.17	0
ALNHCNFGLLVVPIS	0.16	0.16	0.16	0
NLRTNNCATIIVPYI	0.16	0.17	0.16	0
RTNNCATIIVPYINA	0.16	0.17	0.16	0
LRTNNCATIIVPYIN	0.16	0.17	0.16	0
CPHQWINLRTNNCAT	0.16	0.15	0.15	0
PSAEACGYSDRVAQL	0.15	0.11	0.13	0
LLVVPISPLDYDQGA	0.15	0.17	0.16	0
VCPHQWINLRTNNCA	0.15	0.15	0.15	0
SVNRFYTLDTKLWEK	0.15	0.18	0.17	0
VNRFYTLDTKLWEKS	0.15	0.19	0.17	0
AGIPISQLTVCPHQW	0.15	0.18	0.17	0
VSVNRFYTLDTKLWE	0.15	0.19	0.17	0
GQNAQFHYLYRSGFC	0.14	0.15	0.15	0
VGYGEWPSYCSDSDA	0.14	0.06	0.10	0
DVSVNRFYTLDTKLW	0.14	0.18	0.16	0
PDVLTETGVFGQNAQ	0.14	0.13	0.13	0
YCSDSDATAVDKPTR	0.14	0.08	0.11	0
GTVAGGTGTEDSHPP	0.14	0.05	0.09	0
FGQNAQFHYLYRSGF	0.14	0.16	0.15	0
NRFYTLDTKLWEKSS	0.14	0.16	0.15	0
VPISPLDYDQGATPV	0.13	0.09	0.11	0

VDKPTRPDVSVNRFY	0.13	0.08	0.11	0
WKFPDVLTETGVFGQ	0.13	0.13	0.13	0
GIPISQLTVCPHQWI	0.13	0.19	0.16	0
TVCPHQWINLRTNNC	0.13	0.14	0.14	0
TQEAANIIVGYGEWP	0.13	0.12	0.13	0
GYGEWPSYCSDSDAT	0.13	0.06	0.10	0
FPDVLTETGVFGQNA	0.13	0.12	0.13	0
AANIIVGYGEWPSYC	0.13	0.08	0.11	0
IPISQLTVCPHQWIN	0.13	0.18	0.15	0
AVDKPTRPDVSVNRF	0.13	0.07	0.10	0
EAANIIVGYGEWPSY	0.13	0.09	0.11	0
TAVDKPTRPDVSVNR	0.13	0.06	0.09	0
PISQLTVCPHQWINL	0.12	0.17	0.14	0
QEAANIIVGYGEWPS	0.12	0.10	0.11	0
RFYTLDTKLWEKSSK	0.12	0.15	0.14	0
DKPTRPDVSVNRFYT	0.12	0.09	0.11	0
KPTRPDVSVNRFYTL	0.12	0.12	0.12	0
SPSAEACGYSDRVAQ	0.12	0.08	0.10	0
ATAVDKPTRPDVSVN	0.12	0.06	0.09	0
LVVPISPLDYDQGAT	0.12	0.12	0.12	0
PDVSVNRFYTLDTKL	0.12	0.16	0.14	0
LTVCPHQWINLRTNN	0.12	0.13	0.12	0
TVAGGTGTEDSHPPY	0.12	0.05	0.08	0
QLTVCPHQWINLRTN	0.11	0.13	0.12	0
SALNHCNFGLLVVPI	0.11	0.13	0.12	0
ISQLTVCPHQWINLR	0.11	0.16	0.13	0
TQPGADGFELQHPYV	0.11	0.08	0.10	0
TEDSHPPYKQTQPGA	0.11	0.04	0.07	0
SQLTVCPHQWINLRT	0.11	0.13	0.12	0
KFPDVLTETGVFGQN	0.11	0.11	0.11	0
PTRPDVSVNRFYTLD	0.11	0.12	0.11	0
TKLWEKSSKGWYWKF	0.11	0.08	0.09	0
DATAVDKPTRPDVSV	0.11	0.06	0.08	0
SDATAVDKPTRPDVS	0.11	0.06	0.08	0

CSDSDATAVDKPTRP	0.10	0.07	0.09	0
LDTKLWEKSSKGWYW	0.10	0.09	0.10	0
DTKLWEKSSKGWYWK	0.10	0.08	0.09	0
KLWEKSSKGWYWKFP	0.10	0.07	0.08	0
DSALNHCNFGLLVVP	0.10	0.10	0.10	0
TRPDVSVNRFYTLDT	0.09	0.14	0.12	0
FYTLDTKLWEKSSKG	0.09	0.12	0.11	0
VAGGTGTEDSHPPYK	0.09	0.04	0.07	0
VVPISPLDYDQGATP	0.09	0.10	0.10	0
TLDTKLWEKSSKGWY	0.09	0.08	0.09	0
DSDATAVDKPTRPDV	0.09	0.06	0.07	0
RPDVSVNRFYTLDTK	0.09	0.15	0.12	0
SDSDATAVDKPTRPD	0.09	0.06	0.07	0
KQTQPGADGFELQHP	0.09	0.06	0.07	0
WEKSSKGWYWKFPDV	0.09	0.06	0.07	0
QTQPGADGFELQHPY	0.09	0.07	0.08	0
AGGTGTEDSHPPYKQ	0.08	0.04	0.06	0
LWEKSSKGWYWKFPD	0.08	0.06	0.07	0
YTLDTKLWEKSSKGW	0.08	0.09	0.08	0
GGTGTEDSHPPYKQT	0.08	0.04	0.06	0
GTGTEDSHPPYKQTQ	0.08	0.04	0.06	0
TGTEDSHPPYKQTQP	0.07	0.03	0.05	0
GTEDSHPPYKQTQPG	0.06	0.03	0.05	0

[a]H-2-IAb or H-2-IAd are mouse MHCII molecules. 1-log50K indicates the predicted binding affinity of epitope binding to specific MHCII molecule and is presented in log-scale. NB: number of binding.

Supplementary Table 6 The predicted binding potential between a cleaved EV71 VP2 peptide and TCR-MHCI/II complex containing a CASSLGANSDYTF CDR3 sequence

MHC presenting EV71 VP2 peptide	CASSLGANSDYTF
**MHC class I-EV71 VP2 peptide**	
CGYSDRVAQLTI	-694.9[a]
CNASKFHQGALL	–
DSALNHCNFGLL	-864.1
SALNHCNFGLLV	-850.0
**MHC class II-EV71 VP2 peptide**	
–	–

[a]The scores, implemented from ClusPro service, are not suitable to compare the binding energy among peptide-bound TCR-MHCI/II complexes.
